# Supplementary material for: Incidence, severity, and preventability of adverse events during the induction of patients with acute lymphoblastic leukemia in a tertiary care pediatric hospital in Mexico
Source: PLoS One. 2022 Mar 24;17(3):e0265450. doi: 10.1371/journal.pone.0265450 (PMC8947076; doi:10.1371/journal.pone.0265450)
Supplement: S1 Table — (DOCX) [file pone.0265450.s001.docx]

**S1 Table. Incidence of adverse events by patients and admissions during induction therapy.**

|  | **Total**^a^ | **Patients with any adverse event** | | **Total** | **Adverse events per 100 admissions** | |
| --- | --- | --- | --- | --- | --- | --- |
|  |  | **Rate** | **95% CI** |  | **Rate** | **95% CI** |
| Overall | 147 | 81.2 | 75.5 – 86.9 | 172 | 57.7 | 52.1 – 63.3 |
| **Type** |  |  |  |  |  |  |
| Drugs | 145 | 80.1 | 74.3 – 85.9 | 140 | 47.0 | 41.3 – 52.6 |
| Hospital care | 16 | 8.8 | 4.7 – 13.0 | 19 | 6.4 | 3.6 – 9.1 |
| Procedures | 11 | 6.1 | 2.6 – 9.6 | 13 | 4.4 | 2.0 – 6.7 |
| **Severity** |  |  |  |  |  |  |
| Mild | 51 | 28.2 | 21.6 – 34.7 | 18 | 6.0 | 3.3 – 8.7 |
| Moderate | 88 | 48.6 | 41.3 – 55.9 | 58 | 19.5 | 15.0 – 24.0 |
| Severe | 91 | 50.3 | 43.0 – 57.6 | 46 | 15.4 | 11.3 – 19.5 |
| Life-threatening | 47 | 26.0 | 19.6 – 32.4 | 36 | 12.1 | 8.4 – 15.8 |
| Death-related | 18 | 9.9 | 5.6 – 14.3 | 14 | 4.7 | 2.3 – 7.1 |
| **Preventability** |  |  |  |  |  |  |
| Preventable | 31 | 17.1 | 11.6 – 22.6 | 36 | 12.1 | 8.4 – 15.8 |
| Ameliorable | 108 | 59.7 | 52.5 – 66.8 | 47 | 15.8 | 11.6 – 19.9 |
| Non-preventable | 96 | 53.0 | 45.8 – 60.3 | 86 | 28.9 | 23.7 – 34.0 |
| Non-evaluable | 17 | 9.4 | 5.1 – 13.6 | 3 | 1.0 | 0.0 – 2.1 |

Abbreviations. CI: confidence interval. Denominators: 181 patients and 298 hospital admissions since the start of induction.

^a^The sum of total AEs across the type, severity and preventability categories is not equal to 147, because each patient could have suffered more than one AE in each category.
