# Supplementary material for: Incidence, severity, and preventability of adverse events during the induction of patients with acute lymphoblastic leukemia in a tertiary care pediatric hospital in Mexico
Source: PLoS One. 2022 Mar 24;17(3):e0265450. doi: 10.1371/journal.pone.0265450 (PMC8947076; doi:10.1371/journal.pone.0265450)
Supplement: S2 Table — (DOCX) [file pone.0265450.s002.docx]

**S2 Table. Individual description of adverse drug events occurred during remission induction.**

| **Drug** | **Adverse drug events**  **n=367** | **Causality evaluation^†^** | **CTCAE severity** | **Preventability** |
| --- | --- | --- | --- | --- |
| Acetaminophen | Allergic reaction^¶^ | Definite | Moderate | Preventable |
| Acetaminophen | Allergic reaction^¶^ | Definite | Moderate | Preventable |
| Acetaminophen | Allergic reaction | Probable | Moderate | Non-preventable |
| Amphotericin B | Electrolyte disturbance | Probable | Life-threatening | Non-preventable |
| Amphotericin B | Electrolyte disturbance | Probable | Moderate | Non-preventable |
| Amphotericin B | Electrolyte disturbance | Probable | Moderate | Non-preventable |
| Amphotericin B (lipid complex) | Allergic reaction | Probable | Moderate | Non-preventable |
| Amphotericin B (lipid complex) | Electrolyte disturbance | Probable | Severe | Non-preventable |
| Calcium gluconate | Calcinosis cutis | Probable | Severe | Non-preventable |
| Cefepime | Allergic reaction | Probable | Moderate | Non-preventable |
| Cefepime | Allergic reaction | Probable | Moderate | Non-preventable |
| Cefotaxime | Allergic reaction | Probable | Life-threatening | Non-preventable |
| Contumax | Vomiting | Possible | Moderate | Ameliorable |
| Cyclophosphamide | Sepsis | Possible | Death | Ameliorable |
| DAUNOrubicin | Hematuria^¶^ | Possible | Severe | Preventable |
| DAUNOrubicin | Hematuria^¶^ | Possible | Severe | Preventable |
| DAUNOrubicin | Sinus bradycardia^¶^ | Possible | Life-threatening | Preventable |
| DAUNOrubicin | Ventricular arrhythmia | Possible | Severe | Non-preventable |
| DAUNOrubicin | Vomiting^¶^ | Possible | Moderate | Preventable |
| DAUNOrubicin | Vomiting | Possible | Moderate | Ameliorable |
| DAUNOrubicin | Vomiting | Possible | Moderate | Ameliorable |
| DAUNOrubicin | Vomiting | Possible | Moderate | Ameliorable |
| DAUNOrubicin | Vomiting | Possible | Moderate | Ameliorable |
| DAUNOrubicin | Vomiting | Possible | Moderate | Ameliorable |
| DAUNOrubicin | Vomiting | Possible | Moderate | Ameliorable |
| DAUNOrubicin | Vomiting | Possible | Moderate | Ameliorable |
| DAUNOrubicin | Vomiting | Possible | Moderate | Ameliorable |
| DAUNOrubicin | Vomiting^¶^ | Possible | Moderate | Preventable |
| DAUNOrubicin | Anal fistula | Possible | Death | Ameliorable |
| DAUNOrubicin | Anemia | Possible | Severe | Ameliorable |
| DAUNOrubicin | Anemia | Possible | Moderate | Ameliorable |
| DAUNOrubicin | Anemia | Possible | Moderate | Ameliorable |
| DAUNOrubicin | Anemia | Possible | Moderate | Ameliorable |
| DAUNOrubicin | Anemia | Possible | Moderate | Non-evaluable |
| DAUNOrubicin | Anemia | Possible | Moderate | Non-evaluable |
| DAUNOrubicin | Anemia | Possible | Mild | Non-evaluable |
| DAUNOrubicin | Nausea | Possible | Mild | Non-preventable |
| DAUNOrubicin/L-asparaginase | Anemia | Possible | Moderate | Non-evaluable |
| DAUNOrubicin/L-asparaginase | Anemia | Possible | Moderate | Non-evaluable |
| DAUNOrubicin/L-asparaginase | Disseminated intravascular coagulation | Possible | Death | Non-preventable |
| DAUNOrubicin/L-asparaginase | Disseminated intravascular coagulation | Possible | Death | Non-preventable |
| DAUNOrubicin/L-asparaginase | Disseminated intravascular coagulation | Possible | Death | Non-preventable |
| DAUNOrubicin/L-asparaginase | Platelet count decreased | Possible | Severe | Ameliorable |
| DAUNOrubicin/L-asparaginase | Platelet count decreased | Possible | Severe | Ameliorable |
| DAUNOrubicin/L-asparaginase | Platelet count decreased | Possible | Severe | Non-evaluable |
| DAUNOrubicin/L-asparaginase | Platelet count decreased^¶^ | Possible | Moderate | Ameliorable |
| DAUNOrubicin/L-asparaginase | Platelet count decreased | Possible | Moderate | Ameliorable |
| DAUNOrubicin/L-asparaginase | Platelet count decreased | Possible | Moderate | Ameliorable |
| DAUNOrubicin/L-asparaginase | Platelet count decreased | Possible | Moderate | Ameliorable |
| DAUNOrubicin/L-asparaginase | Platelet count decreased | Possible | Moderate | Ameliorable |
| DAUNOrubicin/L-asparaginase | Platelet count decreased | Possible | Mild | Non-evaluable |
| DAUNOrubicin/L-asparaginase | Platelet count decreased^†^ | Possible | Moderate | Ameliorable |
| DAUNOrubicin/VinCRIStine | Lymphocyte count decreased | Possible | Mild | Non-evaluable |
| DAUNOrubicin/VinCRIStine | Neutrophil count decreased | Possible | Severe | Non-evaluable |
| DAUNOrubicin/VinCRIStine | Neutrophil count decreased | Possible | Moderate | Ameliorable |
| DAUNOrubicin/VinCRIStine | Neutrophil count decreased | Possible | Moderate | Ameliorable |
| DAUNOrubicin/VinCRIStine | Neutrophil count decreased | Possible | Mild | Non-evaluable |
| DAUNOrubicin/VinCRIStine | Neutrophil count decreased | Possible | Mild | Non-evaluable |
| DAUNOrubicin/VinCRIStine | Neutrophil count decreased | Possible | Mild | Non-evaluable |
| DAUNOrubicin/VinCRIStine | Neutrophil count decreased | Possible | Mild | Non-evaluable |
| DAUNOrubicin/VinCRIStine/L-asparaginase | Abdominal infection | Possible | Severe | Non-preventable |
| DAUNOrubicin/VinCRIStine/L-asparaginase | Abdominal infection | Possible | Severe | Non-preventable |
| DAUNOrubicin/VinCRIStine/L-asparaginase | Abdominal infection | Possible | Mild | Non-preventable |
| DAUNOrubicin/VinCRIStine/L-asparaginase | Abdominal infection | Possible | Severe | Non-evaluable |
| DAUNOrubicin/VinCRIStine/L-asparaginase | Abdominal infection | Possible | Moderate | Non-evaluable |
| DAUNOrubicin/VinCRIStine/L-asparaginase | Abdominal infection | Possible | Mild | Non-preventable |
| DAUNOrubicin/VinCRIStine/L-asparaginase | Bronchopulmonary hemorrhage^¶^ | Possible | Death | Ameliorable |
| DAUNOrubicin/VinCRIStine/L-asparaginase | Febrile neutropenia | Possible | Life-threatening | Preventable |
| DAUNOrubicin/VinCRIStine/L-asparaginase | Febrile neutropenia | Possible | Life-threatening | Ameliorable |
| DAUNOrubicin/VinCRIStine/L-asparaginase | Febrile neutropenia | Possible | Life-threatening | Ameliorable |
| DAUNOrubicin/VinCRIStine/L-asparaginase | Febrile neutropenia | Possible | Life-threatening | Ameliorable |
| DAUNOrubicin/VinCRIStine/L-asparaginase | Febrile neutropenia | Possible | Life-threatening | Ameliorable |
| DAUNOrubicin/VinCRIStine/L-asparaginase | Febrile neutropenia | Possible | Life-threatening | Ameliorable |
| DAUNOrubicin/VinCRIStine/L-asparaginase | Febrile neutropenia | Possible | Life-threatening | Ameliorable |
| DAUNOrubicin/VinCRIStine/L-asparaginase | Febrile neutropenia | Possible | Life-threatening | Ameliorable |
| DAUNOrubicin/VinCRIStine/L-asparaginase | Febrile neutropenia | Possible | Life-threatening | Ameliorable |
| DAUNOrubicin/VinCRIStine/L-asparaginase | Febrile neutropenia | Possible | Life-threatening | Ameliorable |
| DAUNOrubicin/VinCRIStine/L-asparaginase | Febrile neutropenia | Possible | Life-threatening | Ameliorable |
| DAUNOrubicin/VinCRIStine/L-asparaginase | Febrile neutropenia | Possible | Life-threatening | Ameliorable |
| DAUNOrubicin/VinCRIStine/L-asparaginase | Febrile neutropenia | Possible | Life-threatening | Ameliorable |
| DAUNOrubicin/VinCRIStine/L-asparaginase | Febrile neutropenia | Possible | Life-threatening | Ameliorable |
| DAUNOrubicin/VinCRIStine/L-asparaginase | Febrile neutropenia^¶^ | Possible | Life-threatening | Ameliorable |
| DAUNOrubicin/VinCRIStine/L-asparaginase | Febrile neutropenia^¶^ | Possible | Severe | Preventable |
| DAUNOrubicin/VinCRIStine/L-asparaginase | Febrile neutropenia^¶^ | Possible | Severe | Preventable |
| DAUNOrubicin/VinCRIStine/L-asparaginase | Febrile neutropenia^¶^ | Possible | Severe | Preventable |
| DAUNOrubicin/VinCRIStine/L-asparaginase | Febrile neutropenia^¶^ | Possible | Severe | Preventable |
| DAUNOrubicin/VinCRIStine/L-asparaginase | Febrile neutropenia^¶^ | Possible | Severe | Preventable |
| DAUNOrubicin/VinCRIStine/L-asparaginase | Febrile neutropenia | Possible | Severe | Ameliorable |
| DAUNOrubicin/VinCRIStine/L-asparaginase | Febrile neutropenia^¶^ | Possible | Severe | Ameliorable |
| DAUNOrubicin/VinCRIStine/L-asparaginase | Febrile neutropenia | Possible | Severe | Ameliorable |
| DAUNOrubicin/VinCRIStine/L-asparaginase | Febrile neutropenia | Possible | Severe | Ameliorable |
| DAUNOrubicin/VinCRIStine/L-asparaginase | Febrile neutropenia | Possible | Severe | Ameliorable |
| DAUNOrubicin/VinCRIStine/L-asparaginase | Febrile neutropenia | Possible | Severe | Ameliorable |
| DAUNOrubicin/VinCRIStine/L-asparaginase | Febrile neutropenia | Possible | Severe | Ameliorable |
| DAUNOrubicin/VinCRIStine/L-asparaginase | Febrile neutropenia | Possible | Severe | Ameliorable |
| DAUNOrubicin/VinCRIStine/L-asparaginase | Febrile neutropenia | Possible | Severe | Ameliorable |
| DAUNOrubicin/VinCRIStine/L-asparaginase | Febrile neutropenia | Possible | Severe | Ameliorable |
| DAUNOrubicin/VinCRIStine/L-asparaginase | Febrile neutropenia | Possible | Severe | Ameliorable |
| DAUNOrubicin/VinCRIStine/L-asparaginase | Febrile neutropenia | Possible | Severe | Ameliorable |
| DAUNOrubicin/VinCRIStine/L-asparaginase | Febrile neutropenia | Possible | Severe | Ameliorable |
| DAUNOrubicin/VinCRIStine/L-asparaginase | Febrile neutropenia | Possible | Severe | Ameliorable |
| DAUNOrubicin/VinCRIStine/L-asparaginase | Febrile neutropenia | Possible | Severe | Ameliorable |
| DAUNOrubicin/VinCRIStine/L-asparaginase | Febrile neutropenia | Possible | Severe | Ameliorable |
| DAUNOrubicin/VinCRIStine/L-asparaginase | Febrile neutropenia | Possible | Severe | Ameliorable |
| DAUNOrubicin/VinCRIStine/L-asparaginase | Febrile neutropenia | Possible | Severe | Ameliorable |
| DAUNOrubicin/VinCRIStine/L-asparaginase | Febrile neutropenia | Possible | Severe | Ameliorable |
| DAUNOrubicin/VinCRIStine/L-asparaginase | Febrile neutropenia | Possible | Severe | Ameliorable |
| DAUNOrubicin/VinCRIStine/L-asparaginase | Febrile neutropenia | Possible | Severe | Ameliorable |
| DAUNOrubicin/VinCRIStine/L-asparaginase | Febrile neutropenia | Possible | Severe | Ameliorable |
| DAUNOrubicin/VinCRIStine/L-asparaginase | Febrile neutropenia | Possible | Severe | Ameliorable |
| DAUNOrubicin/VinCRIStine/L-asparaginase | Febrile neutropenia | Possible | Severe | Ameliorable |
| DAUNOrubicin/VinCRIStine/L-asparaginase | Febrile neutropenia | Possible | Severe | Ameliorable |
| DAUNOrubicin/VinCRIStine/L-asparaginase | Febrile neutropenia | Possible | Severe | Ameliorable |
| DAUNOrubicin/VinCRIStine/L-asparaginase | Febrile neutropenia | Possible | Severe | Ameliorable |
| DAUNOrubicin/VinCRIStine/L-asparaginase | Febrile neutropenia | Possible | Severe | Ameliorable |
| DAUNOrubicin/VinCRIStine/L-asparaginase | Febrile neutropenia | Possible | Severe | Ameliorable |
| DAUNOrubicin/VinCRIStine/L-asparaginase | Febrile neutropenia | Possible | Severe | Ameliorable |
| DAUNOrubicin/VinCRIStine/L-asparaginase | Febrile neutropenia | Possible | Severe | Ameliorable |
| DAUNOrubicin/VinCRIStine/L-asparaginase | Febrile neutropenia | Possible | Severe | Ameliorable |
| DAUNOrubicin/VinCRIStine/L-asparaginase | Febrile neutropenia | Possible | Severe | Ameliorable |
| DAUNOrubicin/VinCRIStine/L-asparaginase | Febrile neutropenia | Possible | Severe | Ameliorable |
| DAUNOrubicin/VinCRIStine/L-asparaginase | Febrile neutropenia | Possible | Severe | Ameliorable |
| DAUNOrubicin/VinCRIStine/L-asparaginase | Febrile neutropenia | Possible | Severe | Ameliorable |
| DAUNOrubicin/VinCRIStine/L-asparaginase | Febrile neutropenia | Possible | Severe | Ameliorable |
| DAUNOrubicin/VinCRIStine/L-asparaginase | Febrile neutropenia | Possible | Severe | Ameliorable |
| DAUNOrubicin/VinCRIStine/L-asparaginase | Febrile neutropenia | Possible | Severe | Ameliorable |
| DAUNOrubicin/VinCRIStine/L-asparaginase | Febrile neutropenia | Possible | Severe | Ameliorable |
| DAUNOrubicin/VinCRIStine/L-asparaginase | Febrile neutropenia | Possible | Severe | Ameliorable |
| DAUNOrubicin/VinCRIStine/L-asparaginase | Febrile neutropenia | Possible | Severe | Ameliorable |
| DAUNOrubicin/VinCRIStine/L-asparaginase | Febrile neutropenia | Possible | Severe | Ameliorable |
| DAUNOrubicin/VinCRIStine/L-asparaginase | Febrile neutropenia | Possible | Severe | Ameliorable |
| DAUNOrubicin/VinCRIStine/L-asparaginase | Febrile neutropenia | Possible | Severe | Ameliorable |
| DAUNOrubicin/VinCRIStine/L-asparaginase | Febrile neutropenia | Possible | Severe | Ameliorable |
| DAUNOrubicin/VinCRIStine/L-asparaginase | Febrile neutropenia | Possible | Severe | Ameliorable |
| DAUNOrubicin/VinCRIStine/L-asparaginase | Febrile neutropenia | Possible | Severe | Ameliorable |
| DAUNOrubicin/VinCRIStine/L-asparaginase | Febrile neutropenia | Possible | Severe | Ameliorable |
| DAUNOrubicin/VinCRIStine/L-asparaginase | Febrile neutropenia | Possible | Severe | Ameliorable |
| DAUNOrubicin/VinCRIStine/L-asparaginase | Febrile neutropenia^¶^ | Possible | Life-threatening | Preventable |
| DAUNOrubicin/VinCRIStine/L-asparaginase | Lung infection | Possible | Severe | Non-preventable |
| DAUNOrubicin/VinCRIStine/L-asparaginase | Lung infection | Possible | Severe | Non-preventable |
| DAUNOrubicin/VinCRIStine/L-asparaginase | Mucositis | Possible | Severe | Ameliorable |
| DAUNOrubicin/VinCRIStine/L-asparaginase | Mucositis | Possible | Severe | Ameliorable |
| DAUNOrubicin/VinCRIStine/L-asparaginase | Mucositis | Possible | Severe | Ameliorable |
| DAUNOrubicin/VinCRIStine/L-asparaginase | Mucositis | Possible | Severe | Ameliorable |
| DAUNOrubicin/VinCRIStine/L-asparaginase | Mucositis | Possible | Severe | Ameliorable |
| DAUNOrubicin/VinCRIStine/L-asparaginase | Mucositis | Possible | Severe | Ameliorable |
| DAUNOrubicin/VinCRIStine/L-asparaginase | Mucositis | Possible | Severe | Ameliorable |
| DAUNOrubicin/VinCRIStine/L-asparaginase | Mucositis | Possible | Moderate | Ameliorable |
| DAUNOrubicin/VinCRIStine/L-asparaginase | Mucositis | Possible | Moderate | Ameliorable |
| DAUNOrubicin/VinCRIStine/L-asparaginase | Mucositis | Possible | Moderate | Ameliorable |
| DAUNOrubicin/VinCRIStine/L-asparaginase | Mucositis | Possible | Moderate | Ameliorable |
| DAUNOrubicin/VinCRIStine/L-asparaginase | Mucositis | Possible | Moderate | Ameliorable |
| DAUNOrubicin/VinCRIStine/L-asparaginase | Mucositis | Possible | Moderate | Ameliorable |
| DAUNOrubicin/VinCRIStine/L-asparaginase | Mucositis | Possible | Moderate | Ameliorable |
| DAUNOrubicin/VinCRIStine/L-asparaginase | Mucositis | Possible | Moderate | Ameliorable |
| DAUNOrubicin/VinCRIStine/L-asparaginase | Mucositis | Possible | Mild | Ameliorable |
| DAUNOrubicin/VinCRIStine/L-asparaginase | Mucositis | Possible | Mild | Ameliorable |
| DAUNOrubicin/VinCRIStine/L-asparaginase | Mucositis | Possible | Mild | Ameliorable |
| DAUNOrubicin/VinCRIStine/L-asparaginase | Mucositis^¶^ | Possible | Life-threatening | Preventable |
| DAUNOrubicin/VinCRIStine/L-asparaginase | Multi-organ failure | Possible | Death | Ameliorable |
| DAUNOrubicin/VinCRIStine/L-asparaginase | Multi-organ failure | Possible | Life-threatening | Non-preventable |
| DAUNOrubicin/VinCRIStine/L-asparaginase | Multi-organ failure | Possible | Life-threatening | Non-preventable |
| DAUNOrubicin/VinCRIStine/L-asparaginase | Multi-organ failure | Possible | Life-threatening | Non-preventable |
| DAUNOrubicin/VinCRIStine/L-asparaginase | Multi-organ failure | Possible | Life-threatening | Non-preventable |
| DAUNOrubicin/VinCRIStine/L-asparaginase | Myocardial infarction | Possible | Death | Non-preventable |
| DAUNOrubicin/VinCRIStine/L-asparaginase | Rinovirus infection | Possible | Moderate | Non-preventable |
| DAUNOrubicin/VinCRIStine/L-asparaginase | Sepsis | Possible | Death | Preventable |
| DAUNOrubicin/VinCRIStine/L-asparaginase | Sepsis | Possible | Death | Ameliorable |
| DAUNOrubicin/VinCRIStine/L-asparaginase | Sepsis | Possible | Life-threatening | Ameliorable |
| DAUNOrubicin/VinCRIStine/L-asparaginase | Sepsis | Possible | Life-threatening | Ameliorable |
| DAUNOrubicin/VinCRIStine/L-asparaginase | Sepsis | Possible | Life-threatening | Ameliorable |
| DAUNOrubicin/VinCRIStine/L-asparaginase | Sepsis | Possible | Life-threatening | Ameliorable |
| DAUNOrubicin/VinCRIStine/L-asparaginase | Sepsis | Possible | Life-threatening | Ameliorable |
| DAUNOrubicin/VinCRIStine/L-asparaginase | Sepsis | Possible | Life-threatening | Ameliorable |
| DAUNOrubicin/VinCRIStine/L-asparaginase | Sepsis | Possible | Life-threatening | Ameliorable |
| DAUNOrubicin/VinCRIStine/L-asparaginase | Sepsis | Possible | Life-threatening | Ameliorable |
| DAUNOrubicin/VinCRIStine/L-asparaginase | Sepsis | Possible | Life-threatening | Ameliorable |
| DAUNOrubicin/VinCRIStine/L-asparaginase | Sepsis | Possible | Life-threatening | Ameliorable |
| DAUNOrubicin/VinCRIStine/L-asparaginase | Sepsis | Possible | Life-threatening | Ameliorable |
| DAUNOrubicin/VinCRIStine/L-asparaginase | Sepsis | Possible | Life-threatening | Ameliorable |
| DAUNOrubicin/VinCRIStine/L-asparaginase | Sepsis | Possible | Severe | Ameliorable |
| DAUNOrubicin/VinCRIStine/L-asparaginase | Sepsis | Possible | Severe | Ameliorable |
| DAUNOrubicin/VinCRIStine/L-asparaginase | Sepsis | Possible | Severe | Ameliorable |
| DAUNOrubicin/VinCRIStine/L-asparaginase | Sepsis | Possible | Severe | Ameliorable |
| DAUNOrubicin/VinCRIStine/L-asparaginase | Sepsis | Possible | Severe | Ameliorable |
| DAUNOrubicin/VinCRIStine/L-asparaginase | Sepsis | Possible | Severe | Ameliorable |
| DAUNOrubicin/VinCRIStine/L-asparaginase | Sepsis^¶^ | Possible | Life-threatening | Preventable |
| DAUNOrubicin/VinCRIStine/L-asparaginase | Thrush | Possible | Severe | Ameliorable |
| DAUNOrubicin/VinCRIStine/L-asparaginase | Thrush | Possible | Moderate | Non-preventable |
| DAUNOrubicin/VinCRIStine/L-asparaginase | Varicella | Possible | Moderate | Non-preventable |
| DAUNOrubicin/Amphotericin B | Ventricular arrhythmia | Possible | Moderate | Non-preventable |
| Dexamethasone | Cushingoid | Probable | Mild | Non-preventable |
| Dexamethasone | Cushingoid | Probable | Mild | Non-preventable |
| Dexamethasone | Cushingoid | Probable | Mild | Non-preventable |
| Dexamethasone | Cushingoid | Probable | Mild | Non-preventable |
| Dexamethasone | Electrolyte disturbance | Probable | Severe | Non-preventable |
| Dexamethasone | Electrolyte disturbance | Probable | Severe | Non-preventable |
| Dexamethasone | Gastritis | Probable | Moderate | Non-preventable |
| Dexamethasone | Gastritis | Probable | Moderate | Non-preventable |
| Dexamethasone | Gastritis | Probable | Moderate | Non-preventable |
| Dexamethasone | Gastritis | Probable | Moderate | Non-preventable |
| Dexamethasone | Gastritis | Probable | Mild | Non-preventable |
| Dexamethasone | Hyperglycemia | Probable | Severe | Non-preventable |
| Dexamethasone | Hyperglycemia | Probable | Severe | Non-preventable |
| Dexamethasone | Hyperglycemia^¶^ | Possible | Moderate | Ameliorable |
| Dexamethasone | Hyperglycemia | Probable | Moderate | Non-preventable |
| Dexamethasone | Hyperglycemia | Probable | Moderate | Non-preventable |
| Dexamethasone | Hyperglycemia | Probable | Moderate | Non-preventable |
| Dexamethasone | Hyperglycemia | Probable | Moderate | Non-preventable |
| Dexamethasone | Hyperglycemia | Probable | Moderate | Non-preventable |
| Dexamethasone | Hyperglycemia | Probable | Mild | Non-preventable |
| Dexamethasone | Hyperglycemia | Probable | Mild | Non-preventable |
| Dexamethasone | Hyperglycemia | Probable | Mild | Non-preventable |
| Dexamethasone | Hyperglycemia | Probable | Mild | Non-preventable |
| Dexamethasone | Hyperglycemia | Probable | Mild | Non-preventable |
| Dexamethasone | Hyperglycemia | Probable | Mild | Non-preventable |
| Dexamethasone | Hyperglycemia | Probable | Mild | Non-preventable |
| Dexamethasone | Hyperglycemia | Probable | Mild | Non-preventable |
| Dexamethasone | Hyperglycemia | Probable | Mild | Non-preventable |
| Dexamethasone | Hypertension | Possible | Life-threatening | Non-preventable |
| Dexamethasone | Hypertension | Possible | Moderate | Non-preventable |
| Dexamethasone | Hypertension | Possible | Moderate | Non-preventable |
| Dexamethasone | Hypertension | Possible | Moderate | Non-preventable |
| Dexamethasone | Hypertension | Possible | Moderate | Non-preventable |
| Dexamethasone | Nausea | Possible | Mild | Non-preventable |
| Dexamethasone | Pancreatitis | Possible | Death | Non-preventable |
| Dexamethasone | Skin infection | Possible | Severe | Ameliorable |
| Dexamethasone | Skin infection | Possible | Moderate | Ameliorable |
| Dexamethasone | Skin infection | Possible | Moderate | Non-preventable |
| Dexamethasone | Hyperglycemia | Possible | Moderate | Non-preventable |
| Dexamethasone/L-asparaginase | Gastric hemorrhage | Possible | Severe | Non-preventable |
| Dexamethasone/L-asparaginase | Gastric hemorrhage | Possible | Severe | Non-preventable |
| Dexamethasone/L-asparaginase | Metabolic acidosis | Possible | Life-threatening | Non-preventable |
| Dexamethasone/L-asparaginase | Hyperglycemia | Possible | Severe | Non-preventable |
| Dexamethasone/L-asparaginase | Hyperglycemia | Possible | Moderate | Non-preventable |
| Dexamethasone/L-asparaginase | Hyperglycemia | Possible | Moderate | Non-preventable |
| Dexamethasone/L-asparaginase | Hyperglycemia^¶^ | Possible | Mild | Ameliorable |
| Furosemide | Electrolyte disturbance | Possible | Life-threatening | Non-preventable |
| L-asparaginase | Allergic reaction | Possible | Severe | Non-preventable |
| L-asparaginase | Allergic reaction | Probable | Severe | Non-preventable |
| L-asparaginase | Allergic reaction | Definite | Severe | Non-preventable |
| L-asparaginase | Allergic reaction | Possible | Mild | Non-preventable |
| L-asparaginase | Blood bilirubin increased | Possible | Mild | Non-preventable |
| L-asparaginase | Conjunctival hemorrhage | Possible | Mild | Non-evaluable |
| L-asparaginase | Epistaxis^¶^ | Possible | Moderate | Ameliorable |
| L-asparaginase | Epistaxis^¶^ | Possible | Moderate | Ameliorable |
| L-asparaginase | Epistaxis | Possible | Moderate | Ameliorable |
| L-asparaginase | Epistaxis | Possible | Moderate | Ameliorable |
| L-asparaginase | Epistaxis | Possible | Moderate | Ameliorable |
| L-asparaginase | Fibrinogen decreased | Possible | Mild | Non-preventable |
| L-asparaginase | Hemorrhagic shock | Possible | Death | Ameliorable |
| L-asparaginase | Hepatic failure | Possible | Life-threatening | Non-preventable |
| L-asparaginase | Pancreatitis | Possible | Death | Non-preventable |
| L-asparaginase | Platelet count decreased | Possible | Life-threatening | Ameliorable |
| L-asparaginase | Platelet count decreased | Possible | Severe | Ameliorable |
| L-asparaginase | Platelet count decreased | Possible | Severe | Ameliorable |
| L-asparaginase | Platelet count decreased | Possible | Moderate | Ameliorable |
| L-asparaginase | Platelet count decreased | Possible | Moderate | Ameliorable |
| L-asparaginase | Platelet count decreased | Possible | Moderate | Ameliorable |
| L-asparaginase | Seizure | Possible | Severe | Non-preventable |
| L-asparaginase | Sinus tachycardia | Possible | Moderate | Non-preventable |
| L-asparaginase | Stroke | Possible | Death | Non-preventable |
| L-asparaginase | Stroke | Possible | Life-threatening | Non-preventable |
| L-asparaginase | Stroke | Possible | Life-threatening | Non-preventable |
| L-asparaginase | Stroke | Possible | Severe | Non-preventable |
| L-asparaginase | Stroke | Possible | Severe | Non-preventable |
| L-asparaginase | Stroke | Possible | Death | Non-preventable |
| L-asparaginase | Stroke | Possible | Severe | Non-preventable |
| L-asparaginase | Superficial thrombophlebitis | Possible | Severe | Non-preventable |
| L-asparaginase | Superficial thrombophlebitis | Possible | Moderate | Ameliorable |
| L-asparaginase | Vomiting | Possible | Moderate | Ameliorable |
| L-asparaginase | Vomiting | Possible | Moderate | Ameliorable |
| L-asparaginase | Vomiting | Possible | Moderate | Ameliorable |
| L-asparaginase | Vomiting | Possible | Moderate | Ameliorable |
| L-asparaginase | Vomiting | Possible | Moderate | Ameliorable |
| L-asparaginase | Vomiting | Possible | Moderate | Ameliorable |
| L-asparaginase/Dexamethasone | Pancreatitis | Possible | Severe | Non-preventable |
| L-asparaginase/Dexamethasone | Pancreatitis | Possible | Severe | Non-preventable |
| L-asparaginase/Intrathecal chemotherapy | Seizure | Possible | Life-threatening | Non-preventable |
| L-asparaginase/Intrathecal chemotherapy | Stroke | Possible | Life-threatening | Non-preventable |
| L-asparaginase/VinCRIStine | Hepatic failure | Possible | Life-threatening | Non-preventable |
| L-asparaginase/VinCRIStine | Hepatic failure | Possible | Life-threatening | Non-preventable |
| L-asparaginase/VinCRIStine | Seizure | Possible | Life-threatening | Non-preventable |
| L-asparaginase/VinCRIStine | Seizure | Possible | Life-threatening | Non-preventable |
| L-asparaginase/VinCRIStine | Seizure | Possible | Life-threatening | Non-preventable |
| L-asparaginase/VinCRIStine | Seizure | Possible | Severe | Non-preventable |
| Morphine | Constipation | Probable | Moderate | Ameliorable |
| Morphine | Respiratory depression | Probable | Life-threatening | Ameliorable |
| Packed red blood cells | Allergic reaction | Probable | Life-threatening | Non-preventable |
| Packed red blood cells | Allergic reaction | Probable | Moderate | Non-preventable |
| Packed red blood cells | Allergic reaction | Probable | Moderate | Non-preventable |
| Packed red blood cells | Allergic reaction | Probable | Moderate | Non-preventable |
| Packed red blood cells | Allergic reaction | Probable | Moderate | Non-preventable |
| Packed red blood cells | Allergic reaction | Probable | Moderate | Non-preventable |
| Pooled platelets | Allergic reaction | Probable | Moderate | Non-preventable |
| Pooled platelets | Allergic reaction | Probable | Moderate | Non-preventable |
| Pooled platelets | Allergic reaction | Probable | Moderate | Non-preventable |
| PredniSONE | Hyperglycemia | Probable | Mild | Non-preventable |
| PredniSONE | Hyperglycemia | Probable | Mild | Non-preventable |
| PredniSONE | Metabolic acidosis^¶^ | Possible | Life-threatening | Ameliorable |
| PredniSONE | Pancreatitis^¶^ | Possible | Death | Ameliorable |
| PredniSONE | Vomiting | Possible | Moderate | Ameliorable |
| PredniSONE/Ethinyl Estradiol-Levonorgestrel^‡^ | Hyperglycemia | Possible | Moderate | Non-preventable |
| Propofol/Fentanyl^‡^ | Cardiac dysautonomia | Probable | Life-threatening | Ameliorable |
| Propofol/Fentanyl^‡^ | Sinus bradycardia | Possible | Moderate | Non-preventable |
| Vancomycin | Allergic reaction | Probable | Mild | Non-preventable |
| Vancomycin | Allergic reaction | Probable | Moderate | Non-preventable |
| Vancomycin | Allergic reaction | Probable | Moderate | Non-preventable |
| Vancomycin | Allergic reaction | Probable | Moderate | Non-preventable |
| Vancomycin | Allergic reaction | Probable | Moderate | Non-preventable |
| VinCRIStine | Abdominal pain | Possible | Mild | Non-evaluable |
| VinCRIStine | Abdominal pain | Possible | Mild | Non-evaluable |
| VinCRIStine | Abdominal pain | Possible | Mild | Non-evaluable |
| VinCRIStine | Abdominal pain | Possible | Mild | Non-evaluable |
| VinCRIStine | Abdominal pain | Possible | Mild | Non-evaluable |
| VinCRIStine | Constipation | Possible | Mild | Non-preventable |
| VinCRIStine | Constipation | Possible | Mild | Ameliorable |
| VinCRIStine | Constipation | Possible | Mild | Ameliorable |
| VinCRIStine | Constipation | Possible | Mild | Non-preventable |
| VinCRIStine | Constipation | Possible | Mild | Non-preventable |
| VinCRIStine | Constipation | Possible | Mild | Non-preventable |
| VinCRIStine | Dysesthesia | Probable | Mild | Non-preventable |
| VinCRIStine | Dysesthesia | Probable | Mild | Non-preventable |
| VinCRIStine | Febrile neutropenia^¶^ | Possible | Severe | Preventable |
| VinCRIStine | Febrile neutropenia | Possible | Severe | Ameliorable |
| VinCRIStine | Febrile neutropenia | Possible | Severe | Ameliorable |
| VinCRIStine | Febrile neutropenia | Possible | Severe | Ameliorable |
| VinCRIStine | Headache | Possible | Mild | Non-preventable |
| VinCRIStine | Hearing impaired | Possible | Mild | Non-evaluable |
| VinCRIStine | Ileal perforation | Possible | Life-threatening | Ameliorable |
| VinCRIStine | Ileus^¶^ | Possible | Severe | Ameliorable |
| VinCRIStine | Ileus | Possible | Severe | Non-preventable |
| VinCRIStine | Ileus | Possible | Moderate | Ameliorable |
| VinCRIStine | Ileus | Possible | Moderate | Ameliorable |
| VinCRIStine | Ileus | Possible | Moderate | Ameliorable |
| VinCRIStine | Ileus | Possible | Moderate | Ameliorable |
| VinCRIStine | Ileus | Possible | Mild | Non-preventable |
| VinCRIStine | Mucositis | Possible | Moderate | Ameliorable |
| VinCRIStine | Peripheral neuropathy | Possible | Moderate | Non-preventable |
| VinCRIStine | Peripheral neuropathy | Possible | Moderate | Non-preventable |
| VinCRIStine | Peripheral neuropathy | Possible | Moderate | Non-preventable |
| VinCRIStine | Peripheral neuropathy | Possible | Moderate | Non-preventable |
| VinCRIStine | Peripheral neuropathy | Possible | Moderate | Non-preventable |
| VinCRIStine | Peripheral neuropathy | Possible | Moderate | Non-preventable |
| VinCRIStine | Peripheral neuropathy | Possible | Moderate | Non-preventable |
| VinCRIStine | Peripheral neuropathy | Possible | Moderate | Non-preventable |
| VinCRIStine | Peripheral neuropathy | Possible | Moderate | Non-preventable |
| VinCRIStine | Peripheral neuropathy | Possible | Moderate | Non-preventable |
| VinCRIStine | Peripheral neuropathy | Possible | Moderate | Non-preventable |
| VinCRIStine | Peripheral neuropathy | Possible | Moderate | Non-preventable |
| VinCRIStine | Peripheral neuropathy | Possible | Moderate | Non-preventable |
| VinCRIStine | Peripheral neuropathy | Possible | Moderate | Non-preventable |
| VinCRIStine | Peripheral neuropathy | Possible | Moderate | Non-preventable |
| VinCRIStine | Peripheral neuropathy | Possible | Moderate | Non-preventable |
| VinCRIStine | Peripheral neuropathy | Possible | Moderate | Non-preventable |
| VinCRIStine | Peripheral neuropathy | Possible | Moderate | Non-preventable |
| VinCRIStine | Peripheral neuropathy | Possible | Moderate | Non-preventable |
| VinCRIStine | Peripheral neuropathy | Possible | Mild | Non-preventable |
| VinCRIStine | Peripheral neuropathy | Possible | Mild | Non-preventable |
| VinCRIStine | Peripheral neuropathy | Possible | Mild | Non-preventable |
| VinCRIStine | Peripheral neuropathy | Possible | Mild | Non-preventable |
| VinCRIStine | Seizure | Possible | Life-threatening | Non-preventable |
| VinCRIStine | Sepsis | Possible | Severe | Ameliorable |
| VinCRIStine | Vomiting | Possible | Moderate | Ameliorable |
| VinCRIStine | Vomiting | Possible | Moderate | Ameliorable |
| VinCRIStine | Vomiting | Possible | Moderate | Ameliorable |
| VinCRIStine | Vomiting | Possible | Moderate | Ameliorable |

Abbreviations. CTCAE: Common Terminology Criteria for Adverse Events.

^†^The Naranjo Algorithm was applied to determine adverse drug reactions causality. This causality scale is mandatory for pharmacovigilance activities in Mexico.

^‡^Drug-drug interaction. Identification was carried out using Micromedex^®^ and Up to Date-Lexicomp^®^.

^¶^Adverse drug events related to the presence of medication errors, either with chemotherapy or other drugs that are not part of the remission induction regimen, but that were part of the therapeutic management during the study period (n=27).
