# Supplementary material for: Incidence, severity, and preventability of adverse events during the induction of patients with acute lymphoblastic leukemia in a tertiary care pediatric hospital in Mexico
Source: PLoS One. 2022 Mar 24;17(3):e0265450. doi: 10.1371/journal.pone.0265450 (PMC8947076; doi:10.1371/journal.pone.0265450)
Supplement: S3 Table — (DOCX) [file pone.0265450.s003.docx]

**S3 Table. Adverse event incidence rates by system organ class during induction.**

| **CTCAE system organ class** | **Adverse events**  **n=399** | | **Patient affected**  **n=181** | | **Incidence rate per 1000 patient-days** | |
| --- | --- | --- | --- | --- | --- | --- |
|  | **n** | **%** | **n** | **%** | **Rate** | **95% IC** |
| Blood and lymphatic system disorders^a^ | 95 | 23.8 | 83 | 45.9 | 12.3 | 9.9 – 14.8 |
| Gastrointestinal disorders | 70 | 17.5 | 57 | 31.5 | 9.1 | 7.0 – 11.2 |
| Infections and infestations | 58 | 14.5 | 50 | 27.6 | 7.5 | 5.6 – 9.5 |
| Nervous system disorders | 49 | 12.3 | 42 | 23.2 | 6.4 | 4.6 – 8.1 |
| Metabolism and nutrition disorders | 32 | 8 | 30 | 16.6 | 4.2 | 2.7 – 5.6 |
| Immune system disorders | 25 | 6.3 | 20 | 11.0 | 3.2 | 2.0 – 4.5 |
| Investigations | 19 | 4.8 | 18 | 9.9 | 2.5 | 1.4 – 3.6 |
| Respiratory, thoracic and mediastinal disorders | 14 | 3.5 | 14 | 7.7 | 1.8 | 0.9 – 2.8 |
| Vascular disorders | 9 | 2.3 | 9 | 5.0 | 1.2 | 0.4 – 1.9 |
| Cardiac disorders | 7 | 1.8 | 7 | 3.9 | 0.9 | 0.2 – 1.6 |
| General disorders and administration site conditions | 7 | 1.8 | 7 | 3.9 | 0.9 | 0.2 – 1.6 |
| Endocrine disorders | 4 | 1 | 4 | 2.2 | 0.5 | 0.0 – 1.0 |
| Hepatobiliary disorders | 4 | 1 | 4 | 2.2 | 0.5 | 0.0 – 1.0 |
| Renal and urinary disorders | 2 | 0.5 | 2 | 1.1 | 0.3 | 0.0 – 0.6 |
| Ear and labyrinth disorders | 1 | 0.3 | 1 | 0.6 | 0.1 | 0.0 – 0.4 |
| Eye disorders | 1 | 0.3 | 1 | 0.6 | 0.1 | 0.0 – 0.4 |
| Musculoskeletal and connective tissue disorders | 1 | 0.3 | 1 | 0.6 | 0.1 | 0.0 – 0.4 |
| Skin and subcutaneous tissue disorders | 1 | 0.3 | 1 | 0.6 | 0.1 | 0.0 – 0.4 |

Abbreviations. CTCAE: Common Terminology Criteria for Adverse Events, CI: confidence interval.

^a^CTCAE classify febrile neutropenia as blood and lymphatic system disorders.
