# Supplementary material for: Incidence, severity, and preventability of adverse events during the induction of patients with acute lymphoblastic leukemia in a tertiary care pediatric hospital in Mexico
Source: PLoS One. 2022 Mar 24;17(3):e0265450. doi: 10.1371/journal.pone.0265450 (PMC8947076; doi:10.1371/journal.pone.0265450)
Supplement: S4 Table — (DOCX) [file pone.0265450.s004.docx]

**S4 Table. Adverse events with severity grade ≥3 observed during induction.**

| **CTCAE term** | **Total**  **n=210** | **%** | **Incidence rate per 1000 patient-days** | |
| --- | --- | --- | --- | --- |
|  |  |  | **Rate** | **95% IC** |
| **Infectious adverse events** | | | | |
| Febrile neutropenia | 75 | 35.7 | 9.7 | 7.5 – 11.9 |
| Sepsis | 24 | 11.4 | 3.1 | 1.9 – 4.4 |
| Hospital-acquired infection | 10 | 4.8 | 1.3 | 0.5 – 2.1 |
| Catheter related infection | 3 | 1.4 | 0.4 | 0.0 – 0.8 |
| Abdominal infection | 3 | 1.4 | 0.4 | 0.0 – 0.8 |
| Skin infection | 2 | 1 | 0.3 | 0.0 – 0.6 |
| Lung infection | 2 | 1 | 0.3 | 0.0 – 0.6 |
| Encephalitis infection | 1 | 0.5 | 0.1 | 0.0 – 0.4 |
| Wound infection | 1 | 0.5 | 0.1 | 0.0 – 0.4 |
| Thrush | 1 | 0.5 | 0.1 | 0.0 – 0.4 |
| **Non-infectious adverse events** | | | | |
| Stroke | 9 | 4.3 | 1.2 | 0.4 – 1.9 |
| Seizure | 8 | 3.8 | 1.0 | 0.3 – 1.8 |
| Mucositis | 8 | 3.8 | 1.0 | 0.3 – 1.8 |
| Platelet count decreased | 6 | 2.9 | 0.8 | 0.2 – 1.4 |
| Pancreatitis | 5 | 2.4 | 0.6 | 0.1 – 1.2 |
| Multi-organ failure | 5 | 2.4 | 0.6 | 0.1 – 1.2 |
| Allergic reaction | 5 | 2.4 | 0.6 | 0.1 – 1.2 |
| Electrolyte disturbance | 5 | 2.4 | 0.6 | 0.1 – 1.2 |
| Hepatic failure | 3 | 1.4 | 0.4 | 0.0 – 0.8 |
| Disseminated intravascular coagulation | 3 | 1.4 | 0.4 | 0.0 – 0.8 |
| Hyperglycemia | 3 | 1.4 | 0.4 | 0.0 – 0.8 |
| Metabolic acidosis | 2 | 1 | 0.3 | 0.0 – 0.6 |
| Cerebrospinal fluid leakage | 2 | 1 | 0.3 | 0.0 – 0.6 |
| Gastric hemorrhage | 2 | 1 | 0.3 | 0.0 – 0.6 |
| Ileus | 2 | 1 | 0.3 | 0.0 – 0.6 |
| Hematuria | 2 | 1 | 0.3 | 0.0 – 0.6 |
| Hypertension | 2 | 1 | 0.3 | 0.0 – 0.6 |
| Myocardial infarction | 2 | 1 | 0.3 | 0.0 – 0.6 |
| Superficial thrombophlebitis | 2 | 1 | 0.3 | 0.0 – 0.6 |
| Respiratory depression | 1 | 0.5 | 0.1 | 0.0 – 0.4 |
| Neutrophil count decreased | 1 | 0.5 | 0.1 | 0.0 – 0.4 |
| Hemorrhagic shock | 1 | 0.5 | 0.1 | 0.0 – 0.4 |
| Cardiac dysautonomia | 1 | 0.5 | 0.1 | 0.0 – 0.4 |
| Ileal perforation | 1 | 0.5 | 0.1 | 0.0 – 0.4 |
| Sinus bradycardia | 1 | 0.5 | 0.1 | 0.0 – 0.4 |
| Anal fistula | 1 | 0.5 | 0.1 | 0.0 – 0.4 |
| Anemia | 1 | 0.5 | 0.1 | 0.0 – 0.4 |
| Arachnoiditis | 1 | 0.5 | 0.1 | 0.0 – 0.4 |
| Bronchopulmonary hemorrhage | 1 | 0.5 | 0.1 | 0.0 – 0.4 |
| Calcinosis cutis | 1 | 0.5 | 0.1 | 0.0 – 0.4 |
| Ventricular arrhythmia | 1 | 0.5 | 0.1 | 0.0 – 0.4 |
| **Total** | **210** | **100** | **27.3** | **23.6 – 31.0** |

Abbreviations. CTCAE: Common Terminology Criteria for Adverse Events.
