# Supplementary material for: Incidence, severity, and preventability of adverse events during the induction of patients with acute lymphoblastic leukemia in a tertiary care pediatric hospital in Mexico
Source: PLoS One. 2022 Mar 24;17(3):e0265450. doi: 10.1371/journal.pone.0265450 (PMC8947076; doi:10.1371/journal.pone.0265450)
Supplement: S5 Table — (DOCX) [file pone.0265450.s005.docx]

**S5 Table. Adverse events that required hospital admission during induction.**

| **Adverse event** | **Total**  **n=150** |
| --- | --- |
| Febrile neutropenia | 64 (42.7) |
| Sepsis | 13 (8.7) |
| Mucositis | 13 (8.7) |
| Stroke | 8 (5.3) |
| Vomiting | 7 (4.7) |
| Platelet count decreased | 5 (3.3) |
| Ileus | 5 (3.3) |
| Seizure | 3 (2.0) |
| Hospital-acquired infection | 2 (1.3) |
| Anemia | 2 (1.3) |
| Neutrophil count decreased | 2 (1.3) |
| Abdominal infection | 2 (1.3) |
| Epistaxis | 2 (1.3) |
| Pancreatitis | 2 (1.3) |
| Cerebrospinal fluid leakage | 2 (1.3) |
| Lung infection | 2 (1.3) |
| Metabolic acidosis | 2 (1.3) |
| Hyperglycemia | 1 (0.7) |
| Hypertension | 1 (0.7) |
| Skin infection | 1 (0.7) |
| Hepatic failure | 1 (0.7) |
| Gastric hemorrhage | 1 (0.7) |
| Myocardial infarction | 1 (0.7) |
| Thrush | 1 (0.7) |
| Ventricular arrhythmia | 1 (0.7) |
| Wound infection | 1 (0.7) |
| Anal fistula | 1 (0.7) |
| Calcinosis cutis | 1 (0.7) |
| Encephalitis infection | 1 (0.7) |
| Hemorrhagic shock | 1 (0.7) |
| Rhinovirus infection | 1 (0.7) |
