# Supplementary material for: Incidence, severity, and preventability of adverse events during the induction of patients with acute lymphoblastic leukemia in a tertiary care pediatric hospital in Mexico
Source: PLoS One. 2022 Mar 24;17(3):e0265450. doi: 10.1371/journal.pone.0265450 (PMC8947076; doi:10.1371/journal.pone.0265450)
Supplement: S6 Table — (DOCX) [file pone.0265450.s006.docx]

**S6 Table. Adverse events frequency by severity and preventability during induction.**

| **Adverse events** | **Total**  **n=399** | **CTCAE Severity** | | | | | | **Preventability** | | | | |
| --- | --- | --- | --- | --- | --- | --- | --- | --- | --- | --- | --- | --- |
|  |  | **Mild**  **n=57** | **Moderate**  **n=132** | **Severe**  **n=128** | **Life-threatening**  **n=64** | **Death-related**  **n=18** | | **Preventable**  **n=42** | **Ameliorable**  **n=177** | **Non-preventable**  **n=157** | **Non-evaluable**  **n=23** | |
| Febrile neutropenia | 75 (18.8) | 0 | 0 | 59 | 16 | 0 | | 8 | 67 | 0 | 0 | |
| Allergic reaction | 25 (6.3) | 2 | 18 | 3 | 2 | 0 | | 2 | 0 | 23 | 0 | |
| Hyperglycemia | 25 (6.3) | 12 | 10 | 3 | 0 | 0 | | 0 | 2 | 23 | 0 | |
| Sepsis | 24 (6.0) | 0 | 0 | 7 | 14 | 3^a^ | | 2 | 22 | 0 | 0 | |
| Vomiting | 24 (6.0) | 0 | 24 | 0 | 0 | 0 | | 2 | 20 | 2 | 0 | |
| Peripheral neuropathy | 23 (5.8) | 4 | 19 | 0 | 0 | 0 | | 0 | 0 | 23 | 0 | |
| Mucositis | 20 (5.0) | 3 | 9 | 7 | 1 | 0 | | 1 | 19 | 0 | 0 | |
| Platelet count decreased | 16 (4.0) | 1 | 9 | 5 | 1 | 0 | | 0 | 14 | 0 | 2 | |
| Hospital-acquired infection | 10 (2.5) | 0 | 0 | 7 | 2 | 1^a^ | | 10 | 0 | 0 | 0 | |
| Anemia | 9 (2.3) | 1 | 7 | 1 | 0 | 0 | | 0 | 4 | 0 | 5 | |
| Stroke | 9 (2.3) | 0 | 0 | 3 | 4 | 2 | | 0 | 0 | 9 | 0 | |
| Seizure | 8 (2.0) | 0 | 0 | 2 | 6 | 0 | | 0 | 0 | 8 | 0 | |
| Constipation | 7 (1.8) | 6 | 1 | 0 | 0 | 0 | | 0 | 3 | 4 | 0 | |
| Electrolyte abnormalities | 7 (1.8) | 0 | 2 | 3 | 2 | 0 | | 0 | 0 | 7 | 0 | |
| Ileus | 7 (1.8) | 1 | 4 | 2 | 0 | 0 | | 0 | 5 | 2 | 0 | |
| Neutrophil count decreased | 7 (1.8) | 4 | 2 | 1 | 0 | 0 | | 0 | 2 | 0 | 5 | |
| Abdominal infection | 6 (1.5) | 2 | 1 | 3 | 0 | 0 | | 0 | 0 | 4 | 2 | |
| Hypertension | 6 (1.5) | 0 | 4 | 1 | 1 | 0 | | 0 | 0 | 6 | 0 | |
| Abdominal pain | 5 (1.3) | 5 | 0 | 0 | 0 | 0 | | 0 | 0 | 0 | 5 | |
| Epistaxis | 5 (1.3) | 0 | 5 | 0 | 0 | 0 | | 0 | 5 | 0 | 0 | |
| Gastritis | 5 (1.3) | 1 | 4 | 0 | 0 | 0 | | 0 | 0 | 5 | 0 | |
| Multi-organ failure | 5 (1.3) | 0 | 0 | 0 | 4 | 1^a^ | | 0 | 1 | 4 | 0 | |
| Pancreatitis | 5 (1.3) | 0 | 0 | 2 | 0 | 3 | | 0 | 1 | 4 | 0 | |
| Skin infection | 5 (1.3) | 1 | 2 | 2 | 0 | 0 | | 1 | 2 | 1 | 1 | |
| Catheter-related infection | 5 (1.3) | 0 | 2 | 3 | 0 | | 0 | 5 | 0 | 0 | 0 |  |
| Cushingoid | 4 (1.0) | 4 | 0 | 0 | 0 | | 0 | 0 | 0 | 4 | 0 |  |
| Cerebrospinal fluid leakage | 3 (0.8) | 0 | 1 | 2 | 0 | | 0 | 3 | 0 | 0 | 0 |  |
| Disseminated intravascular coagulation | 3 (0.8) | 0 | 0 | 0 | 0 | | 3^a^ | 0 | 0 | 3 | 0 |  |
| Hepatic failure | 3 (0.8) | 0 | 0 | 0 | 3 | | 0 | 0 | 0 | 3 | 0 |  |
| Superficial thrombophlebitis | 3 (0.8) | 0 | 1 | 2 | 0 | | 0 | 1 | 1 | 1 | 0 |  |
| Dysesthesia | 2 (0.5) | 2 | 0 | 0 | 0 | | 0 | 0 | 0 | 2 | 0 |  |
| Gastric hemorrhage | 2 (0.5) | 0 | 0 | 2 | 0 | | 0 | 0 | 0 | 2 | 0 |  |
| Hematuria | 2 (0.5) | 0 | 0 | 2 | 0 | | 0 | 2 | 0 | 0 | 0 |  |
| Lung infection | 2 (0.5) | 0 | 0 | 2 | 0 | | 0 | 0 | 0 | 2 | 0 |  |
| Metabolic acidosis | 2 (0.5) | 0 | 0 | 0 | 2 | | 0 | 0 | 1 | 1 | 0 |  |
| Myocardial infarction | 2 (0.5) | 0 | 0 | 0 | 0 | | 2 | 1 | 0 | 1 | 0 |  |
| Nausea | 2 (0.5) | 2 | 0 | 0 | 0 | | 0 | 0 | 0 | 2 | 0 |  |
| Sinus bradycardia | 2 (0.5) | 0 | 1 | 0 | 1 | | 0 | 1 | 0 | 1 | 0 |  |
| Thrush | 2 (0.5) | 0 | 1 | 1 | 0 | | 0 | 0 | 1 | 1 | 0 |  |
| Ventricular arrhythmia | 2 (0.5) | 0 | 1 | 1 | 0 | | 0 | 0 | 0 | 2 | 0 |  |
| Wound infection | 2 (0.5) | 0 | 1 | 1 | 0 | | 0 | 2 | 0 | 0 | 0 |  |
| Anal fistula | 1 (0.3) | 0 | 0 | 0 | 0 | | 1 | 0 | 1 | 0 | 0 |  |
| Arachnoiditis | 1 (0.3) | 0 | 0 | 0 | 1 | | 0 | 0 | 1 | 0 | 0 |  |
| Blood bilirubin increased | 1 (0.3) | 1 | 0 | 0 | 0 | | 0 | 0 | 0 | 1 | 0 |  |
| Bronchopulmonary hemorrhage | 1 (0.3) | 0 | 0 | 0 | 0 | | 1 | 0 | 1 | 0 | 0 |  |
| Calcinosis cutis | 1 (0.3) | 0 | 0 | 1 | 0 | | 0 | 0 | 0 | 1 | 0 |  |
| Cardiac dysautonomia | 1 (0.3) | 0 | 0 | 0 | 1 | | 0 | 0 | 1 | 0 | 0 |  |
| Conjunctival hemorrhage | 1 (0.3) | 1 | 0 | 0 | 0 | | 0 | 0 | 0 | 0 | 1 |  |
| Encephalitis infection | 1 (0.3) | 0 | 0 | 0 | 1 | | 0 | 1 | 0 | 0 | 0 |  |
| Fibrinogen decreased | 1 (0.3) | 1 | 0 | 0 | 0 | | 0 | 0 | 0 | 1 | 0 |  |
| Headache | 1 (0.3) | 1 | 0 | 0 | 0 | | 0 | 0 | 0 | 1 | 0 |  |
| Hearing impaired | 1 (0.3) | 1 | 0 | 0 | 0 | | 0 | 0 | 0 | 0 | 1 |  |
| Hemorrhagic shock | 1 (0.3) | 0 | 0 | 0 | 0 | | 1 | 0 | 1 | 0 | 0 |  |
| Ileal perforation | 1 (0.3) | 0 | 0 | 0 | 1 | | 0 | 0 | 1 | 0 | 0 |  |
| Lymphocyte count decreased | 1 (0.3) | 1 | 0 | 0 | 0 | | 0 | 0 | 0 | 0 | 1 |  |
| Respiratory depression | 1 (0.3) | 0 | 0 | 0 | 1 | | 0 | 0 | 1 | 0 | 0 |  |
| Rhinovirus infection | 1 (0.3) | 0 | 1 | 0 | 0 | | 0 | 0 | 0 | 1 | 0 |  |
| Sinus tachycardia | 1 (0.3) | 0 | 1 | 0 | 0 | | 0 | 0 | 0 | 1 | 0 |  |
| Varicella | 1 (0.3) | 0 | 1 | 0 | 0 | | 0 | 0 | 0 | 1 | 0 |  |

Abbreviations. CTCAE: Common Terminology Criteria for Adverse Events.

^a^Deaths related to infectious processes during induction therapy.
