# Supplementary material for: Incidence, severity, and preventability of adverse events during the induction of patients with acute lymphoblastic leukemia in a tertiary care pediatric hospital in Mexico
Source: PLoS One. 2022 Mar 24;17(3):e0265450. doi: 10.1371/journal.pone.0265450 (PMC8947076; doi:10.1371/journal.pone.0265450)
Supplement: S7 Table — (DOCX) [file pone.0265450.s007.docx]

**S7 Table. Preventability of adverse events and their causes.**

| **Type of adverse event** | **Total**  **n=399** | **Preventability – n (%)** | | | |
| --- | --- | --- | --- | --- | --- |
|  |  | **Preventable**  **n=42** | **Ameliorable**  **n=177** | **Non-preventable**  **n=157** | **Non-evaluable**  **n=23** |
| **Drugs** | 367 | 18 (42.9) | 175 (98.9) | 152 (96.8) | 22 (95.7) |
| Adverse drug reaction | 340 | 4 (9.5) | 165 (93.2) | 149 (94.9) | 22 (95.7) |
| Medication error | 27 | 14 (33.3) | 10 (5.6) | 3 (1.9) | 0 |
| **Hospital care** | 19 | 18 (42.9) | 0 | 0 | 1 (4.3) |
| Hospital-acquired infections. | 17 | 17 (40.5) | 0 | 0 | 0 |
| Others | 2 | 1 (2.4) | 0 | 0 | 1 (4.3) |
| **Procedures** | 13 | 6 (14.3) | 2 (1.1) | 5 (3.2) | 0 |
| Lumbar puncture | 11 | 4 (9.5) | 2 (1.1) | 5 (3.2) | 0 |
| Catheter insertion | 2 | 2 (4.8) | 0 | 0 | 0 |
