# Supplementary material for: Incidence, severity, and preventability of adverse events during the induction of patients with acute lymphoblastic leukemia in a tertiary care pediatric hospital in Mexico
Source: PLoS One. 2022 Mar 24;17(3):e0265450. doi: 10.1371/journal.pone.0265450 (PMC8947076; doi:10.1371/journal.pone.0265450)
Supplement: S8 Table — (DOCX) [file pone.0265450.s008.docx]

**S8 Table. Adverse events weekly frequency during remission induction**

| **Adverse events** | **Follow-up weeks^†^** | | | | | | | | | | | **Total** |
| --- | --- | --- | --- | --- | --- | --- | --- | --- | --- | --- | --- | --- |
|  | **1** | **2** | **3** | **4** | **5** | **6** | **7** | **8** | **9** | **10** | **11** |  |
| **Drug-related** |  |  |  |  |  |  |  |  |  |  |  |  |
| Febrile neutropenia | 0 | 12 | 14 | 18 | 7 | 11 | 7 | 2 | 3 | 0 | 1 | 75 |
| Allergic reaction | 10 | 3 | 1 | 2 | 3 | 2 | 2 | 1 | 1 | 0 | 0 | 25 |
| Hyperglycemia | 12 | 5 | 4 | 2 | 0 | 0 | 1 | 1 | 0 | 0 | 0 | 25 |
| Peripheral neuropathy | 0 | 3 | 2 | 6 | 7 | 2 | 2 | 1 | 0 | 0 | 0 | 23 |
| Sepsis | 0 | 4 | 1 | 8 | 6 | 2 | 1 | 0 | 1 | 0 | 0 | 23 |
| Vomiting | 2 | 7 | 1 | 2 | 5 | 2 | 2 | 0 | 1 | 0 | 0 | 22 |
| Mucositis | 0 | 4 | 5 | 3 | 3 | 3 | 2 | 0 | 0 | 0 | 0 | 20 |
| Platelet count decreased | 0 | 5 | 3 | 7 | 1 | 0 | 0 | 0 | 0 | 0 | 0 | 16 |
| Anemia | 0 | 4 | 1 | 2 | 0 | 2 | 0 | 0 | 0 | 0 | 0 | 9 |
| Stroke | 0 | 2 | 1 | 1 | 1 | 3 | 0 | 0 | 0 | 0 | 0 | 8 |
| Constipation | 0 | 1 | 3 | 1 | 0 | 1 | 1 | 0 | 0 | 0 | 0 | 7 |
| Electrolyte disturbance | 2 | 1 | 0 | 1 | 3 | 0 | 0 | 0 | 0 | 0 | 0 | 7 |
| Ileus | 0 | 2 | 1 | 1 | 3 | 0 | 0 | 0 | 0 | 0 | 0 | 7 |
| Neutrophil count decreased | 0 | 1 | 1 | 2 | 3 | 0 | 0 | 0 | 0 | 0 | 0 | 7 |
| Seizure | 0 | 0 | 1 | 0 | 3 | 1 | 1 | 0 | 0 | 1 | 0 | 7 |
| Abdominal infection | 0 | 0 | 0 | 4 | 2 | 0 | 0 | 0 | 0 | 0 | 0 | 6 |
| Abdominal pain | 0 | 0 | 0 | 1 | 3 | 0 | 0 | 0 | 0 | 1 | 0 | 5 |
| Epistaxis | 0 | 0 | 1 | 1 | 0 | 2 | 0 | 1 | 0 | 0 | 0 | 5 |
| Gastritis | 1 | 1 | 1 | 1 | 1 | 0 | 0 | 0 | 0 | 0 | 0 | 5 |
| Hypertension | 2 | 0 | 0 | 1 | 1 | 1 | 0 | 0 | 0 | 0 | 0 | 5 |
| Multi-organ failure | 0 | 0 | 0 | 1 | 2 | 1 | 0 | 1 | 0 | 0 | 0 | 5 |
| Pancreatitis | 1 | 0 | 2 | 0 | 1 | 1 | 0 | 0 | 0 | 0 | 0 | 5 |
| Cushingoid | 0 | 0 | 1 | 0 | 0 | 2 | 1 | 0 | 0 | 0 | 0 | 4 |
| Disseminated intravascular coagulation | 0 | 0 | 0 | 0 | 2 | 1 | 0 | 0 | 0 | 0 | 0 | 3 |
| Hepatic failure | 0 | 0 | 0 | 2 | 0 | 1 | 0 | 0 | 0 | 0 | 0 | 3 |
| Skin infection | 1 | 0 | 0 | 0 | 1 | 1 | 0 | 0 | 0 | 0 | 0 | 3 |
| Dysesthesia | 0 | 0 | 0 | 2 | 0 | 0 | 0 | 0 | 0 | 0 | 0 | 2 |
| Gastric hemorrhage | 0 | 1 | 0 | 1 | 0 | 0 | 0 | 0 | 0 | 0 | 0 | 2 |
| Hematuria | 0 | 2 | 0 | 0 | 0 | 0 | 0 | 0 | 0 | 0 | 0 | 2 |
| Lung infection | 0 | 0 | 0 | 0 | 1 | 1 | 0 | 0 | 0 | 0 | 0 | 2 |
| Metabolic acidosis | 0 | 2 | 0 | 0 | 0 | 0 | 0 | 0 | 0 | 0 | 0 | 2 |
| Nausea | 1 | 0 | 0 | 1 | 0 | 0 | 0 | 0 | 0 | 0 | 0 | 2 |
| Sinus bradycardia | 0 | 1 | 0 | 0 | 1 | 0 | 0 | 0 | 0 | 0 | 0 | 2 |
| Superficial thrombophlebitis | 1 | 0 | 0 | 0 | 0 | 1 | 0 | 0 | 0 | 0 | 0 | 2 |
| Thrush | 0 | 0 | 0 | 0 | 0 | 1 | 1 | 0 | 0 | 0 | 0 | 2 |
| Ventricular arrhythmia | 0 | 0 | 0 | 0 | 0 | 2 | 0 | 0 | 0 | 0 | 0 | 2 |
| Anal fistula | 0 | 0 | 0 | 0 | 0 | 0 | 1 | 0 | 0 | 0 | 0 | 1 |
| Blood bilirubin increased | 0 | 1 | 0 | 0 | 0 | 0 | 0 | 0 | 0 | 0 | 0 | 1 |
| Bronchopulmonary hemorrhage | 0 | 0 | 0 | 0 | 0 | 0 | 0 | 0 | 1 | 0 | 0 | 1 |
| Calcinosis cutis | 0 | 0 | 0 | 0 | 0 | 0 | 1 | 0 | 0 | 0 | 0 | 1 |
| Cardiac dysautonomia | 1 | 0 | 0 | 0 | 0 | 0 | 0 | 0 | 0 | 0 | 0 | 1 |
| Conjunctival hemorrhage | 0 | 0 | 0 | 0 | 1 | 0 | 0 | 0 | 0 | 0 | 0 | 1 |
| Fibrinogen decreased | 0 | 1 | 0 | 0 | 0 | 0 | 0 | 0 | 0 | 0 | 0 | 1 |
| Headache | 0 | 0 | 0 | 1 | 0 | 0 | 0 | 0 | 0 | 0 | 0 | 1 |
| Hearing impaired | 0 | 0 | 0 | 0 | 0 | 0 | 0 | 1 | 0 | 0 | 0 | 1 |
| Hemorrhagic shock | 0 | 0 | 0 | 1 | 0 | 0 | 0 | 0 | 0 | 0 | 0 | 1 |
| Ileal perforation | 0 | 0 | 0 | 0 | 1 | 0 | 0 | 0 | 0 | 0 | 0 | 1 |
| Lymphocyte count decreased | 0 | 1 | 0 | 0 | 0 | 0 | 0 | 0 | 0 | 0 | 0 | 1 |
| Myocardial infarction | 0 | 0 | 0 | 1 | 0 | 0 | 0 | 0 | 0 | 0 | 0 | 1 |
| Respiratory depression | 0 | 0 | 0 | 0 | 1 | 0 | 0 | 0 | 0 | 0 | 0 | 1 |
| Rinovirus infection | 0 | 0 | 0 | 1 | 0 | 0 | 0 | 0 | 0 | 0 | 0 | 1 |
| Sinus tachycardia | 0 | 0 | 0 | 0 | 1 | 0 | 0 | 0 | 0 | 0 | 0 | 1 |
| Varicella | 0 | 0 | 0 | 0 | 0 | 1 | 0 | 0 | 0 | 0 | 0 | 1 |
| **Hospital care-related** |  |  |  |  |  |  |  |  |  |  |  |  |
| Hospital-acquired infection | 1 | 1 | 0 | 1 | 1 | 3 | 1 | 1 | 1 | 0 | 0 | 10 |
| Catheter related infection | 2 | 0 | 2 | 0 | 1 | 0 | 0 | 0 | 0 | 0 | 0 | 5 |
| Skin infection | 0 | 0 | 0 | 0 | 2 | 0 | 0 | 0 | 0 | 0 | 0 | 2 |
| Wound infection | 0 | 0 | 0 | 0 | 2 | 0 | 0 | 0 | 0 | 0 | 0 | 2 |
| **Procedure-related** |  |  |  |  |  |  |  |  |  |  |  |  |
| Cerebrospinal fluid leakage | 0 | 0 | 0 | 1 | 0 | 1 | 1 | 0 | 0 | 0 | 0 | 3 |
| Vomiting | 1 | 0 | 0 | 0 | 1 | 0 | 0 | 0 | 0 | 0 | 0 | 2 |
| Arachnoiditis | 0 | 0 | 0 | 0 | 1 | 0 | 0 | 0 | 0 | 0 | 0 | 1 |
| Encephalitis infection | 0 | 0 | 0 | 0 | 0 | 1 | 0 | 0 | 0 | 0 | 0 | 1 |
| Hypertension | 1 | 0 | 0 | 0 | 0 | 0 | 0 | 0 | 0 | 0 | 0 | 1 |
| Myocardial infarction | 0 | 0 | 0 | 0 | 1 | 0 | 0 | 0 | 0 | 0 | 0 | 1 |
| Seizure | 0 | 0 | 0 | 0 | 1 | 0 | 0 | 0 | 0 | 0 | 0 | 1 |
| Sepsis | 0 | 0 | 0 | 0 | 0 | 0 | 0 | 1 | 0 | 0 | 0 | 1 |
| Stroke | 0 | 0 | 0 | 0 | 1 | 0 | 0 | 0 | 0 | 0 | 0 | 1 |
| Superficial thrombophlebitis | 1 | 0 | 0 | 0 | 0 | 0 | 0 | 0 | 0 | 0 | 0 | 1 |
| **Total** | 40 | 65 | 46 | 77 | 75 | 50 | 25 | 10 | 8 | 2 | 1 | 399 |

^†^Ideal correspondence between follow-up weeks and the remission induction protocol:

Week 1: usually diagnosis and clinical stabilization.

Week 2: steroid window begins.

Week 3: first week of induction chemotherapy: Vincristine/Daunorubicin/L-asparaginase x3.

Week 4: second week of induction chemotherapy: Vincristine/Daunorubicin/L-asparaginase x3.

Week 5: third week of induction chemotherapy: Vincristine/Daunorubicin/L-asparaginase x3.

Week 6: fourth week of induction chemotherapy: Vincristine/Corticosteroid gradual reduction.

Week 7-11: weeks in which remission induction therapy continues in patients who experienced a delay due to adverse events. Hospitalization delays can occur on any follow-up week.
