# Supplementary material for: Incidence, severity, and preventability of adverse events during the induction of patients with acute lymphoblastic leukemia in a tertiary care pediatric hospital in Mexico
Source: PLoS One. 2022 Mar 24;17(3):e0265450. doi: 10.1371/journal.pone.0265450 (PMC8947076; doi:10.1371/journal.pone.0265450)
Supplement: S1 File — (PDF) [file pone.0265450.s009.pdf]

Hospital Infantil de México Federico Gómez

Departamento de Evaluación de Medicamentos y Farmacovigilancia

Protocolo de investigación

Incidencia, severidad y prevenibilidad de eventos adversos en el tratamiento de inducción a la remisión de pacientes con leucemia linfoblástica aguda en un hospital pediátrico de tercer nivel en México.

Fecha: 27 de agosto de 2021

Versión corregida.

Índice

INTRODUCCIÓN 4

MARCO TEÓRICO 5

Calidad de la atención y Seguridad del paciente. 5

Eventos adversos en atención a la salud. 5

Medición de los eventos adversos. 5

Causalidad y tipos de eventos adversos. 6

Eventos adversos asociados a procedimientos y cirugías. 6

Eventos adversos asociados a medicamentos. 7

Infecciones asociadas a la atención hospitalaria 7

Severidad. 8

Prevenibilidad. 8

ANTECEDENTES 9

Leucemia linfoblástica aguda en México. 9

Atención de la leucemia linfoblástica aguda y seguridad del paciente. 9

Eventos adversos en oncología y la prevenibilidad. 10

PLANTEAMIENTO DEL PROBLEMA 15

PREGUNTA DE INVESTIGACIÓN 16

JUSTIFICACIÓN 16

OBJETIVOS 16

MÉTODOS 17

Diseño de estudio. 17

Población de estudio. 17

Marco muestral. 17

Unidad de muestreo....... 17
Criterios de selección........ 18
Diseño de la muestra. 19
Variables de estudio 19
Procedimiento. 24
Diseño estadístico........ 27
Consideraciones éticas y de bioseguridad 28
REFERENCIAS 29

INTRODUCCIÓN

Los eventos adversos constituyen un daño al paciente asociado con el manejo médico, en lugar de complicaciones por una enfermedad subyacente. Algunos eventos adversos aparecen por fallas en los procesos de atención que podrían ser prevenibles. De tal modo que el estudio de la frecuencia e impacto de los eventos adversos prevenibles se considera necesario para mejorar la seguridad del paciente y la calidad de la atención.

Durante el manejo médico de los pacientes con leucemia linfoblástica aguda, la presencia de eventos adversos relacionados a la terapia que reciben se traduce en una pesada carga de síntomas para ellos, particularmente en poblaciones pediátricas donde la frecuencia y severidad del daño puede subestimarse.

En México como otros países de ingresos medios y bajos, los protocolos de tratamiento de leucemia linfoblástica aguda se adaptan de protocolos exitosos en países con alto nivel de desarrollo y menores limitaciones de recursos en sus sistemas de salud. Por consiguiente es necesario investigar la seguridad de tales adaptaciones para identificar oportunidades de reducción y prevención de daño al paciente.

Sin embargo, en nuestro país no se realizado una descripción de los eventos adversos con un enfoque en la prevenibilidad del daño asociado al cuidado de la salud. Por ello, el objetivo de este protocolo es estudiar la frecuencia, tipo, severidad y prevenibilidad de los eventos adversos ocurridos en pacientes pediátricos con leucemia linfoblástica aguda, durante la fase de inducción a la remisión de pacientes atendidos en un Instituto Nacional de Salud pediátrico.

MARCO TEÓRICO

Calidad de la atención y Seguridad del paciente.

La Organización Mundial de la Salud (OMS) define calidad de la atención como el grado en que la atención para los sujetos y poblaciones incrementan la probabilidad de obtener un resultado de salud deseado[1]. Una de las dimensiones que constituyen una atención de calidad es la seguridad del paciente[1,2]. Esta es un tema central ya que una atención insegura puede resultar en daño a los pacientes y costos económicos para los sistemas de salud[3]. La OMS define seguridad del paciente como la ausencia de daño prevenible durante el proceso de atención y la reducción de riesgos de daño innecesario, relacionados con la atención sanitaria hasta un mínimo aceptable con base en el conocimiento actual, los recursos disponibles, y el contexto de la atención[4].

Eventos adversos en atención a la salud.

Se estima que uno de cada cuatro pacientes en los países de ingresos medios y bajos sufre algún evento adverso (EA) en hospitalización como resultado de la atención recibida[5]. Además se estima que 15% del consumo hospitalario en países de ingresos altos se utiliza en atender EA por una atención insegura[6]. Existen diversas definiciones de EA, todas involucran la presencia de un daño o signo desfavorable en el paciente como consecuencia del manejo médico y no por una enfermedad subyacente o su progreso[7-9]. También se suele enfatizar la relación temporal del EA con algún tratamiento médico o procedimiento[10,11], así como el carácter involuntario del daño[7,12].

Es bien sabido que la aplicación de la medicina clínica implica una relación intrínseca entre beneficio a la salud y riesgo de daño al paciente, tal como en los procedimientos quirúrgicos o la utilización de medicamentos; pero también las fallas en los procesos, sistemas o equipos que intervienen en cualquier etapa del cuidado de la salud pueden resultar en daños para los pacientes[9]. En el primer caso se denominan EA no prevenibles, en el segundo caso se denominan EA prevenibles[13].

Medición de los eventos adversos.

Los EA se consideran indicadores útiles en la evaluación de la seguridad del paciente, por lo cual existen distintos métodos para su medición, como son: los sistemas de reporte voluntario anónimo, la identificación prospectiva, y la revisión retrospectiva de registros clínicos. Este

último método se considera el método estándar de búsqueda y evaluación epidemiológica de EA[14], debido a su mayor eficiencia en comparación con los métodos de reporte voluntario y el menor consumo de recursos y tiempo de estudio que los métodos de medición prospectivos. Sin embargo, la principal desventaja de las revisiones retrospectivas es la calidad y completitud de los registros clínicos, la necesidad de exhaustividad y experiencia de los revisores, así como la necesidad de revisar grandes volúmenes de registros[15].

Debido que ningún método único puede identificar todos los EA ocurridos en la atención de los pacientes[16,17], se suelen combinar distintos métodos, así como herramientas complementarias para hacer más eficiente la búsqueda prospectiva o retrospectiva de EA[18].

En los métodos retrospectivos participan al menos dos revisores experimentados en la atención a pacientes, altamente calificados en la aplicación de los mejores estándares de la medicina clínica. Ambos deben realizar una revisión independiente y cegada de cada registro clínico, tanto para la determinación de cada EA, causalidad, así como severidad y prevenibilidad. Las evaluaciones sin acuerdo entre los revisores suelen ser resueltas por la revisión de un tercer revisor experto, o la discusión de los casos entre los revisores para alcanzar un consenso; pero cuando este no se alcanza, la decisión la toma un tercer juez[19,20].

Causalidad y tipos de eventos adversos.

La evaluación de la causalidad y severidad son necesarias para cualquier EA porque ayudan a determinar su importancia en el ámbito clínico[21]. Adicionalmente, otros atributos como servicio de aparición, especialidad de atención, y especialmente la prevenibilidad suelen ser de importancia para describir el desempeño de los sistemas de atención[15,22,23].

La clasificación por etiología distingue comúnmente tres grandes tipos: (i) EA post operatorios o complicaciones relacionadas a procedimientos médicos o reingresos a cirugía, (ii) EA asociados a medicamentos (EAM), (iii) y EA por infecciones asociadas a la atención hospitalaria (infecciones nosocomiales) [22]. Otros EA con frecuencia variable de acuerdo al ámbito de atención y población atendida son los EA asociados a diagnóstico, caídas o fracturas, anestesia, posparto u obstétrico, entre otros[15,22].

Eventos adversos asociados a procedimientos y cirugías.

La ejecución poco segura de procedimientos quirúrgicos provoca EA por complicaciones hasta en 25% de los pacientes[24]. Se consideran EA de este tipo aquellos daños ocasionados por fallas en la ejecución técnica o planeación de un procedimiento invasivo bajo anestesia general, regional o sedación profunda, como existencia de cuerpo extraño, sangrado postoperatorio, quemadura, lesión en órganos, lesión vascular u otra de naturaleza no infecciosa[25-27]. En poblaciones pediátricas mexicanas de 0 a 16 años, 1 de cada 10 pacientes puede presentar un EA relacionado a procedimientos quirúrgicos, entre las cuales 29% son complicaciones por lesión en órgano, falla en la aplicación técnica de un procedimiento, retraso en el tratamiento, y cuerpos extraños[25]. Los EA prevenibles más frecuentes están relacionados con la técnica y sangrado postoperatorio, que en conjunto pueden ser prevenibles en 74%[26,28].

Eventos adversos asociados a medicamentos.

Un EAM es el daño causado por un medicamento, ya sea su uso apropiado o inapropiado[9,11,20,29]. La definición tradicional de EAM incluye: respuestas nociva al fármaco, no intencionadas, que se presentan con dosis normalmente usadas para la profilaxis, diagnóstico, tratamiento de una enfermedad o modificación de una función fisiológica en el ser humano que normalmente se denominan reacciones adversas a medicamentos (RAMs); también interacciones farmacológicas manifestadas clínicamente, toxicidad aguda o falta de eficacia terapéutica por uso inapropiado o errores de medicación (EM) (dosificación, prescripción inapropiada, notas ilegibles, etc), y abuso de los medicamentos[20,30].

Infecciones asociadas a la atención hospitalaria

Las infecciones contraídas por un paciente durante su tratamiento en un hospital u otra institución de atención, y que el paciente no estaba incubando al momento de su ingreso se denominan infecciones asociadas al cuidado hospitalario[31]. Su concepto se extiende a infecciones asociadas a la atención sanitaria en sentido amplio[32]. También son uno de los EA más frecuentes durante la hospitalización de un paciente con impacto en la mortalidad[33] y se consideran fallas del sistema que se asocian con mayor número de complicaciones, duración y costo de la estancia hospitalaria[13,34], y han sido más estudiadas en adultos que en niños[34]. Varios factores de riesgo contribuyen en su aparición, como una técnica deficiente de colocación de sondas, catéteres u otros dispositivos médicos invasivos, pacientes con traumas, quemaduras e inmunodeprimidos, así como fallas en las prácticas básicas de higiene[13].

Severidad.

El termino severidad describe la intensidad de un evento clínico sea cual sea su etiología[19]. Medir la severidad de un EA permite comunicar su relevancia clínica, mientras que estandarizar las escalas permite hacer más confiable la información sobre seguridad del paciente, proyectos de mejora de la calidad y ayuda a establecer la razón riesgo/beneficio de las terapias usadas habitualmente[21]. La intensidad se mide por escalas con criterios explícitos para cada categoría, y que pueden ser genéricas o específicas para un grupo de pacientes o especialidad[35,36].

Para clasificar los EA en pacientes oncológicos se utiliza habitualmente la *Common Terminology Criteria for Adverse Events (CTCAE)* [37-40], una taxonomía desarrollada por el *National Cancer Institute* de Estados Unidos, cuya quinta versión clasifica la severidad de cientos de EA mediante criterios clínicos y marcadores biomédicos específicos, los cuales se dividen en cinco grados de intensidad[36]:

Grado 1 (leve): observaciones clínicas o diagnósticas, signos de laboratorio asintomáticos o síntomas leves que no requieren intervención médica.

Grado 2 (moderado): manifestaciones que requieren intervenciones mínimas, locales o no invasivas.

Grado 3 (severo): requieren o prolongan la hospitalización o son incapacitantes.

Grado 4 (amenaza la vida): cuando se requiere una intervención médica urgente.

Grado 5 (muerte): cuando la muerte está relacionada al EA.

Prevenibilidad.

Aunque el concepto de prevenibilidad no tiene una definición en consenso[41], se considera que un EA es prevenible cuando los métodos para evitar el daño se conocen[42], es decir, cuando es resultado de una atención clínica inconsistente con un estándar de práctica aceptado, o una complicación relacionada a un tratamiento que pudo ser anticipada[43]. Por esto, se necesita distinguir el daño prevenible del que no lo es[13,37], pues algunos pacientes experimentan daño aun recibiendo la mejor terapia[44] bajo el principio de justicia donde el beneficio supere al riesgo de acción o no acción[45]. Pero tampoco la presencia de fallas o errores en el manejo médico conduce necesariamente a la aparición de algún EA, y no todos los errores son completamente prevenibles[46].

Por lo anterior, se han propuesto diversos instrumentos para determinar la prevenibilidad de los EA, cuya estructura, método y criterios de evaluación difieren entre sí[41]. Ciertos tipos de instrumentos requieren la aplicación subjetiva del criterio del evaluador, buscando aplicar el concepto de adherencia a la mejor práctica médica[7,47,48]. Todos estos ofrecen una definición de prevenibilidad, algunos incluyen una escala tipo Likert o criterios específicos para determinar grados de prevenibilidad. Se consideran el mejor método para evaluar prevenibilidad, porque permiten evaluar múltiples factores y el contexto del EA. Sin embargo, requiere de un alto nivel de experiencia clínica y capacitación profesional[20,41]. Otros instrumentos están basados en algoritmos de decisión[49] y tienen un enfoque reduccionista de la prevenibilidad, así como una capacidad limitada para captar la complejidad de la atención, siendo sumamente influenciables por el sesgo retrospectivo[41].

ANTECEDENTES

Leucemia linfoblástica aguda en México.

En México la principal enfermedad neoplásica en grupos pediátricos es la leucemia linfoblástica aguda (LLA)[50,51]. Es el tipo más común de leucemias agudas en la niñez, y se caracteriza por una proliferación desregulada de las células precursoras de linfocitos en la médula ósea[51,52]. En América Latina la mortalidad temprana relacionada al tratamiento de la LLA en pediatría no se ha reducido por décadas[53,54] y particularmente en México, donde la mortalidad durante la primera fase de tratamiento, la inducción a la remisión, varía desde 5.4% hasta 15%[55,56].

Atención de la leucemia linfoblástica aguda y seguridad del paciente.

Los protocolos modernos de quimioterapia para LLA tienen tres etapas fundamentales: la inducción a la remisión, consolidación y mantenimiento[52,57,58]. Entre estas la inducción a la remisión es una etapa crítica para la seguridad de los pacientes, porque las complicaciones de la actividad de la leucemia siguen manifestándose, mientras que se presentan por primera vez EA asociados al manejo médico de la enfermedad[59,60]. La recurrencia conocida de dichos EA y complicaciones se traduce en una pesada carga de síntomas para los pacientes[61]. Adicionalmente, se ha mostrado que se subestima la frecuencia y severidad de los EA, particularmente en

pacientes de oncología pediátrica debido a limitaciones en la comunicación de los niños, la mala interpretación de los familiares o la subestimación de signos o síntomas por parte de los oncólogos[10,62].

Se debe considerar que en México como en otros países de ingresos bajos y medios, los protocolos de quimioterapia aplicados suelen adaptarse de protocolos exitosos desarrollados en países de ingresos altos[53,63], donde los sistemas de salud disponen de mayores recursos para la atención y el contexto socioeconómico de sus poblaciones es sustancialmente distinto[64]. Sumado a la escasez de intervenciones eficaces para el tratamiento o mitigación para todos los EA que se asocian al manejo terapéutico de los pacientes en sus diferentes aspectos[61]. Debido a lo anterior, se considera fundamental hacer énfasis en la investigación de EA[23,65-67] e identificar oportunidades de prevención de daño al paciente, para optimizar el éxito de tales adaptaciones[53,63] y la seguridad de los procesos de atención[68-70].

Eventos adversos en oncología y la prevenibilidad.

Pocos estudios han investigado la prevenibilidad de diversos tipos de EA en pacientes oncológicos, ya sea adultos o pediátricos[12,68,71-75]. Otros estudio sólo se han enfocado en las características de EAM[37,76-78]. La mayor cantidad de estudios enfocados en la prevenibilidad de EA provienen de países de ingresos medios y bajos, en los cuales predominan métodos de evaluación explícitos basados en algoritmos que priorizan un enfoque reduccionista de los procesos de atención[73-78].

A nivel internacional, los EA en grupos pediátricos pueden aparecer entre 1.3% y 3.0% de las hospitalizaciones[79]. Específicamente la tasa de incidencia de EA observada entre pacientes oncológicos hospitalizados varía en el rango de 2.3 a 106 EA cada 1000 días-paciente[12,68,71-73,75]. La amplia variación no sólo aparece entre estudio de países de ingresos altos, medio y bajos, sino también dentro de países de ingresos altos[79].

Por su parte, la proporción de EA prevenibles también son ampliamente variables entre grupos diversos de pacientes oncológicos, variando entre 7 y 50.7%[12,37,68,71-75]. Las principales fuentes de variación son el tipo de fármacos quimioterapéuticos predominantes de un estudio a otro, así como el uso de escalas con criterios explícitos o algoritmos de decisión y con criterios implícitos

o subjetivos de expertos, estos últimos suelen tener mayor validez al tiempo que representan mayor tiempo y preparación que otros métodos.

Cuadro 1. Publicaciones sobre eventos adversos en pacientes oncológicos que incluyen el análisis de la prevenibilidad.									
Autor/ año	Diseño	Muestra	Evaluación de EA			Severidad		Prevenibilidad	
			Método revisión	Causalidad	Frecuencia	Instrumento	Proporción	Instrumento	Frecuencia
Chopra, 2016[73] India	Cohorte prospectiva	Pacientes con carcinomas tratados en un hospital de tercer nivel	Reporte espontaneo de EA	Escala de probabilidad de Naranjo	IA= 58.6%	Escala Hartwig Sigel	0.2% severos<br>12.9% moderados<br>87% leves	Criterios Schumock-Thornton (Explícito)	7% prevenibles<br>42% probablemente prevenibles<br>51% no prevenibles
Shrestha, 2017[75] Nepal	Cohorte prospectiva	Pacientes con cáncer que recibieron quimioterapia y que presentaron al menos un EA	Vigilancia activa	Escala de probabilidad de Naranjo	n.a.	Escala Hartwig Sigel	6.1% severos<br>73.4 moderados<br>20.5% leves	Criterios Schumock-Thornton (Explícito)	42.7% prevenibles<br>52.2% probablemente prevenibles<br>5.1% no prevenibles
Lau, 2004[74] Australia	Cohorte retro-prospectiva	Pacientes >18 años atendidos en un Centro Especializado que aceptaron participar	Vigilancia activa y revisión de expedientes	Escala de probabilidad de Naranjo	IA= 74.3%	n.a.	n.a.	Criterios Schumock-Thornton (Explícito)	53.4% no prevenible<br>45% probablemente prevenibles<br>2% prevenibles
Lipitz, 2017[12] EEUU	Cohorte retrospectiva	Adultos >18a con cáncer de mama, colorrectal o pulmón del NCI	Revisión de expedientes en dos etapas con GTT	Revisión de expertos	TI=2.3/1000 dp	NCCMERP	94% serios<br>6% daño permanente	Criterios de Ghandi (Implícito)	31.5 EA/1000dp prevenibles<br>3.8 EA/1000dp mitigables<br>55.9 EA/1000dp no prevenibles
Call, 2014[37] EEUU	Cohorte retrospectiva	Pacientes con cancer atendidos en el St. Jude Children´s Research Hospital	Reporte espontaneo de EAM y revisión de registros electrónicos en dos etapas con Canadian Trigger Tool	Escala del HMPS	n.a.	NCCMERP y CTCAE v4	82% daño temporal<br>3% daño permanente<br>3% pone en peligro la vida	Escala de prevenibilidad del Canadian Trigger Tool (Implícito)	36.3% prevenible<br>15.2% probablemente prevenible<br>12.1% posiblemente prevenible<br>27.2% probablemente no prevenible<br>9.1% no prevenible
Gerber,	Cohorte	Pacientes >18	Revisión de	Revisión y	IA=42%	NCCMERP	67.4% requirió	Definición de	22% prevenibles

2020[68]<br>Suiza	retrospectiva	años egresados de tres unidades oncológicas	expedientes y análisis de datos con GTT adaptada	acuerdo por pares	TI=107 EA/1000dp<br><br>75.4 EA/100 ingresos		intervención médica<br>30.3% daño temporal<br>2.2%	Hiatt (Implícito)	17% indeterminados<br>61% no prevenibles
Nazer, 2014[71,72]<br>Jordania	Cohorte prospectiva	Pacientes oncológicos adultos en terapia intensiva de un hospital de tercer nivel	Seguimiento día a día por con GTT por un intensivista, farmacéutico y oncólogo. Reporte voluntario anónimo. Y Análisis en dos etapas.	Escala de probabilidad de Naranjo	TI=96.5 EA/1000dp<br><br>35.3 EA/100 ingresos	Criterios propios.	64.8% ponen en riesgo la vida<br>34.1% significativos<br>1.1% fatales	Acuerdo en revisión independiente por dos intensivistas. (Implícito)	15.9%-17.5% prevenibles
Stockwell, 2018[80]<br>EEUU	Cohorte retrospectiva	Pacientes pediátricos de 16 hospitales en 4 regiones geográficas de EEUU	Análisis de datos electrónicos con GAPPS en dos etapas.	Revisión y acuerdo por pares.	TI=19 EA/1000dp<br><br>10.9 EA/100 ingresos	NCCMERP	52.7% requirió intervención médica<br>35.3% daño temporal<br>1.2% daño permanente<br>10.1% pone en peligro la vida<br>0.7% fatales	Criterios propios en revisión independiente por pares a partir de una escala tipo Likert de cuatro puntos. (implícito)	50.7% prevenibles
Wahlang JB, 2017[76]<br>India	Cohorte propspectiva	Pacientes oncológicos de un hospital de tercer nivel	Vigilancia activa y revisión de expedientes	Escala de probabilidad de Naranjo	IA=58.8% EAM	Escala Hartwig Sigel	77.4% leves<br>18.9% moderados<br>3.8% severos	Criterios Schumock-Thornton (Explícito)	45.3% prevenibles<br>54.7% no prevenibles
Ramasubbu, 2020[77]<br>India	Cohorte propspectiva	Pacientes recién diagnosticados con cáncer hematológico y no hematológico que recibieron quimioterapia en un hospital de	Vigilancia activa y revisión de expedientes	Escala de probabilidad de Naranjo	IA=46.6% EAM	Escala Hartwig Sigel	11.6% leves<br>80.2% moderados<br>8.2% severos	Criterios Schumock-Thornton (Explícito)	13.2% prevenibles<br>13.6% probablemente prevenibles<br>73.2% no prevenibles

		tercer nivel.							
Sharma, 2015[78]	Cohorte retrospectiva	Pacientes oncológicos en un hospital de tercer nivel recibiendo quimioterapia que presentaron EAM	Revisión de expedientes por oncólogos y residentes.	Escala de probabilidad de Naranjo	n.r.	Escala Hartwig Sigel	30.6% leves<br>61.4% moderadas<br>6% severo	Criterios Schumock-Thornton (Explícito)	30.8% prevenible<br>3.8% probablemente prevenibles<br>65.4% no prevenible
Abreviaturas. CTCAE: Common Terminology Criteria for Adverse Events, dp: días-paciente, EA: eventos adversos, EEUU: Estados Unidos, GTT: global trigger tool, HMPS: Harvard Medical Practice Study, IA, incidencia acumulada, n.a.: no aplica, NCCMERP: National Coordinating Council for Medication Error Reporting and Prevention, n.r: no reportado, NCI: National Cancer Institute, TI: tasa de incidencia,									

PLANTEAMIENTO DEL PROBLEMA

Los pacientes pediátricos recién diagnosticados con LLA sufren frecuentemente de EA asociados al manejo médico del cáncer. Estos EA representan una pesada carga física y psicológica para los pacientes y sus familias. Además, en México estos pacientes presentan una mortalidad elevada durante la fase de inducción a la remisión de quimioterapia, lo cual es motivo de atención porque no se ha reducido por décadas.

En la actualidad se busca poner mayor énfasis en la calidad de la atención que reciben estos pacientes. Se ha mostrado que durante la fase de inducción a la remisión ocurren fallas en el sistema de medicación que comprometen la seguridad de quimioterapia y pueden contribuir en la aparición de EAM prevenibles hasta en 12.2% de los pacientes en esta fase de terapia, algunos de los cuales podrían contribuir en la mortalidad. Pero también otros procesos de atención pueden producir daño involuntario sobre el paciente cuando no se cumplen los mejores estándares de calidad y seguridad alcanzables en el contexto en que se presta la atención, como los procedimientos médicos invasivos o quirúrgicos, así como deficiencias en el cuidado y prevención de infecciones adquiridas en el hospital, los cuales son prioridades para la seguridad del paciente.

Sin embargo, en México no hay estudios que evalúen la prevenibilidad, severidad y frecuencia de los EA que se presentan en pacientes pediátricos con LLA. A nivel internacional, pocos estudios se han enfocado en la descripción de la prevenibilidad de los EA entre pacientes con enfermedades oncológicas, y sus estimaciones son ampliamente variables por las diferencias en el diseño de los estudios, la disponibilidad de múltiples instrumentos para medir la prevenibilidad de EA, pero sobre todo los tipos de pacientes y regímenes de quimioterapia predominantes de un estudio a otro. Por lo cual es necesario que se estudien los patrones locales, a fin de identificar procesos de atención con posibilidades de mejorar la seguridad del paciente y la calidad de los servicios hacia este grupo de pacientes.

PREGUNTA DE INVESTIGACIÓN

¿Cuál es la incidencia de los EA asociados al cuidado de la salud por su tipo, severidad y prevenibilidad, que se presentan en pacientes pediátricos con LLA durante la terapia de inducción a la remisión en el Hospital Infantil de México Federico Gómez (HIMFG)?

JUSTIFICACIÓN

Este estudio epidemiológico sobre EA permitirá identificar la proporción prevenible del daño asociado al manejo médico de la LLA que se produce sobre pacientes pediátricos durante la inducción a la remisión. El enfoque centrado en la prevenibilidad de los EA busca aspectos del cuidado donde los mejores estándares de atención alcanzables presentan deficiencias en su aplicación u organización, y proponer medidas de mejora de la calidad y la seguridad del paciente.

La inducción a la remisión es una fase crítica en la terapia contra LLA, en la cual se manifiesta la actividad de la enfermedad al mismo tiempo que se experimenta la carga de efectos citotóxicos e inmunosupresores de la quimioterapia, y en cuyo periodo se concentra una elevada tasa de mortalidad asociada al tratamiento en México y América Latina. Por lo cual, estimar la fracción prevenible del daño al paciente, permitirá identificar oportunidades de reducción de la morbilidad y mortalidad asociada al manejo de su enfermedad, traduciéndose en mejoras sobre la seguridad de los pacientes y la mejora en los servicios de atención.

OBJETIVOS

Objetivo primario

Estimar la incidencia de los EA asociados al cuidado de la salud por su tipo, severidad y prevenibilidad, que se presentan en pacientes pediátricos con LLA durante la terapia de inducción a la remisión en el HIMFG.

Objetivos secundarios

- Determinar la presencia de EA asociados al cuidado de la salud durante la terapia de inducción a la remisión de pacientes pediátricos con LLA en el HIMFG.
- Clasificar los EA asociados al cuidado de la salud en tres tipos: asociados a medicamentos, asociados al cuidado hospitalario, y asociados a procedimientos médicos o quirúrgicos.
- Determinar el grado de severidad de los EA identificados en la cohorte.
- Determinar el grado de prevenibilidad de los EA identificados en la cohorte.

MÉTODOS.

Diseño de estudio.

Cohorte retrolectiva de expedientes clínicos.

Población de estudio.

Pacientes pediátricos con LLA diagnosticados y atendidos en el HIMFG.

Marco muestral.

Expedientes clínicos de pacientes pediátricos con LLA diagnosticados y tratados en el HIMFG durante la fase de inducción a la remisión, entre el 01 de enero de 2015 y el 31 de diciembre de 2017 que formaron parte del estudio HIM-2018-018, "Evaluación de Errores de Medicación durante el Tratamiento de Inducción a la Remisión en Pacientes Pediátricos con Leucemia Linfoblástica Aguda", el cual contó con la aprobación de los Comités de Investigación, Ética y Bioseguridad del HIMFG.

Unidad de muestreo.

Expedientes clínicos de pacientes con LLA.

Criterios de selección.

Criterios de inclusión:

- Registros clínicos de pacientes menores de 18 años
- Diagnosticados con LLA *de novo* entre el 01 de enero de 2015 y el 31 de diciembre de 2017
- Que cuenten con información de manejo médico desde la fecha de primer ingreso por sospecha de LLA hasta el día 28 de inducción a la remisión o la defunción del paciente.
- Que cuenten con los registros fechados al menos del 90% de los fármacos quimioterapéuticos y no quimioterapéuticos prescritos y administrados durante la inducción a la remisión (excluyendo soluciones fisiológicas sin electrolitos de alto riesgo).
- Que cuenten con registros fechados y descripciones legibles de las intervenciones quirúrgicas o procedimiento invasivo que requirieron anestesia local o general y que hayan sido realizados dentro del HIMFG como parte del manejo de LLA.
- Registros completos y consistentemente fechados, en mínimo 90% de las notas de evolución en hospitalización, interconsultas de especialidades, notas de consulta ambulatoria, así como resultados impresos de exámenes clínicos y paraclínicos realizados durante la inducción a la remisión.

Criterios de exclusión:

- Registros clínicos de pacientes sin diagnóstico concluyente de LLA o con diagnósticos distintos.
- Registros clínicos de pacientes con LLA diagnosticados en el HIMFG que fueron trasladados a otras instituciones para su manejo durante la inducción a la remisión.
- Registros incompletos o truncos por abandono del paciente.

- Registros no encontrados en el departamento de bioestadística del HIMFG.
- Registros de pacientes con diagnóstico de LLA que fallecen por complicaciones propias de la LLA antes de iniciar la ventana de esteroides con dexametasona o prednisona.
- Pacientes previamente libres de la enfermedad que presentan recaída de LLA y reciben esquemas de reinducción a la remisión de muy alto riesgo.

Diseño de la muestra.

Método de muestreo: no probabilístico a conveniencia.

Tamaño de muestra: se incluyen todos los registros de pacientes en la cohorte que cumplan criterios de inclusión en el periodo de estudio buscando maximizar la potencia estadística.

Variables de estudio

Cuadro 2. Definición de variables.	
Evento adverso	**Definición conceptual**: Daño o perjuicio no intencionado relacionado temporalmente al manejo médico, en lugar de complicaciones por el avance de una enfermedad subyacente[7-9].<br>**Definición operacional**: Daño ocurrido al paciente identificado en registros clínicos y determinado por expertos como asociado al cuidado de la salud. Se pueden observar más de un EA por cada paciente en distintos momentos durante la inducción a la remisión.<br>**Tipo de variable**: Cualitativa.<br>**Escala de variable**: Nominal dicotómica (presente/ausente).
Tipo de evento adverso.	**Definición conceptual**: Clasificación basada en la causa asociada al EA.<br>**Definición operacional**: Se distinguirán tres tipos de EA en función de su causa asociada:<br>– EAM: respuestas nocivas documentadas hacia un medicamento utilizado con fines profilácticos, terapéuticos o diagnósticos (incluyendo RAMs) y que pueden ocurrir en presencia de EM en cualquier etapa del proceso de medicación.<br>– EA asociados a procedimientos o cirugías: cuando se documente algún tipo de daño durante la ejecución técnica de un procedimiento invasivo planeado[81,82] (por ejemplo: punciones lumbares, inserción de catéteres centrales, aspirados de médula ósea, cirugías).

	– EA asociados al cuidado hospitalario: cuando se documenten daños por falta de cumplimiento de estándares internacionales de seguridad del paciente durante el manejo hospitalario (por ejemplo: caídas, infecciones adquiridas en el hospital).<br>**Tipo de variable**: Cualitativa.<br>**Escala de variable**: Nominal politómica.
Severidad	**Definición conceptual**: Intensidad de un evento clínico independientemente de su etiología[19].<br>**Definición operacional:** El grado asignado por revisores expertos aplicando los criterios de la CTCAE vesión 5[36] desarrollados por el el *National Cancer Institute*. Se podrá asignar el nivel más alto alcanzado por cada EA identificado en cinco grados de severidad:<br>– Grado 1 (leve): observaciones clínicas o diagnósticas, signos de laboratorio asintomáticos o síntomas leves que no requieren intervención médica.<br>– Grado 2 (moderado): manifestaciones que requieren intervenciones mínimas, locales o no invasivas.<br>– Grado 3 (severo): requieren o prolongan la hospitalización o son incapacitantes.<br>– Grado 4 (amenaza la vida): cuando se requiere una intervención médica urgente.<br>– Grado 5 (mortal): cuando la muerte está relacionada al EA.<br>**Tipo de variable**: Cualitativa.<br>**Escala de variable**: Ordinal.
Prevenibilidad	**Definición conceptual:** Daño que se puede evitar siguiendo un estándar de práctica médica en base al estado de conocimiento actual[43].<br>**Definición operacional**: Se aplicará la escala y criterios adaptados de Gandhi[130,40,41] para determinar el grado de prevenibilidad de los EA observados por revisores expertos en oncología pediátrica. Se distinguen tres categorías originales y una cuarta adaptada: EA prevenibles, EA mitigables, EA no prevenibles y EA no evaluables.<br>– Un AE se considerará prevenible si fue resultado de una atención clínica inconsistente con la práctica oncológica estándar, o como una complicación relacionada con el tratamiento que pudo ser anticipada de acuerdo con el estado de la evidencia conocida.<br>– Se considerarán EA mitigables o mejorables aquellos AE cuya severidad o duración se pudo haber reducido considerablemente, si se hubieran tomado acciones distintas, incluyendo aquellas que están más allá de las posibilidades de atención actuales del hospital.<br>– Los EA no evaluables serán aquellos EA cuyo contexto no sea suficientemente detallado en los registros, o se carezca de información importante para que los expertos emitan un juicio sobre su prevenibilidad.<br>– Los EA no prevenibles serán todos aquellos que no cumplan ninguno de los criterios anteriores.<br>**Tipo de variable**: Cualitativa.

	**Escala de variable**: Ordinal.
Covariables	
Sexo	**Definición conceptual:** Condición orgánica que distingue al varón de la mujer.<br>**Definición operacional:** se tomará el sexo registrado en la base de datos y que proviene del expediente clínico particular de cada paciente.<br>**Tipo de variable:** Cualitativa<br>**Escala de medición:** Nominal dicotómica (masculino/femenino)
Grupo de edad	**Definición conceptual:** Etapa de desarrollo que comprende el tiempo de vida transcurrido entre el nacimiento y el momento del diagnóstico.<br>**Definición operacional:** la edad en años, meses y días cumplidos registrada en la base de datos y asentada en la nota médica inicial del expediente al momento del diagnóstico será transformada en categorías en base a los siguientes criterios:<br>– Neonatos: 0 a 28 días.<br>– Infantes: 29 días a < 2.0 años.<br>– Niños: ≥ 2.0 años a <12.0 años<br>– Adolescentes: ≥ 12.0 años a <18.0 años<br>**Tipo de variable:** Cualitativa<br>**Escala de medición:** Ordinal.
Inmunofenotipo	**Definición conceptual:** Tipo de célula diferenciada de origen linfoide que distingue a la leucemia en el torrente sanguíneo.<br>**Definición operacional:** Se consignará el fenotipo reportado en el expediente obtenido de la prueba de inmunofenotipo realizada al momento del diagnóstico de la leucemia y registrada en la base de datos, la cual comprende las siguientes:<br>– Linfocitos B<br>– Linfocitos T<br>– Mixto o indefinido<br>**Tipo de variable:** Cualitativa.<br>**Escala de medición:** Nominal politómica.
Conteo de glóbulos blancos	**Definición conceptual:** Es el número de glóbulos blancos en sangre denominados leucocitos.<br>**Definición operacional:** Se recogerá el número de leucocitos por unidad de volumen expresado en número de células $x10^9/L$ y reportado en el expediente clínico o en la biometría hemática al momento del diagnóstico. El valor registrado en la base de datos será transformado de escala cuantitativa continua a cuatro categorías tal como sigue:<br>– $< 10\ x10^9/L$<br>– 10 a $49.99\ x10^9/L$<br>– 50 a $99.99\ x10^9/L$<br>– $\geq 100\ x10^9/L$<br>**Tipo de variable:** Cualitativa.<br>**Escala de medición:** Ordinal.
Clasificación de riesgo	**Definición conceptual**: Clasificación de riesgo que guía la intensidad del tratamiento de inducción a la remisión.

	**Definición operacional:** se utilizará la clasificación de riesgo desarrollada por el *National Cancer Institute* con los datos registrados al momento del diagnóstico de acuerdo con los siguientes criterios:<br>– Riesgo habitual: edad de 1 a 9.9 años, conteo inicial de glóbulos blancos < 50 x10^9/L.<br>– Riesgo alto: edad ≥10 años y <1 año, conteo inicial de glóbulos blancos ≥ 50 x10^9/L.<br>**Tipo de variable:** Cualitativa.<br>**Medición:** Nominal dicotómica.
Morfología celular de la leucemia	***Definición conceptual**: La caracterización morfológica predominante de las células leucémicas.*<br>***Definición operacional**: Se consignará la morfología celular reportada en el expediente como parte de la caracterización de la leucemia: L1, L2, L3.*<br>***Tipo de variable**: Cualitativa*<br>***Escala de medición**: Nominal politómica.*
Citogenética (transcriptos moleculares)	***Definición conceptual**: Alteraciones genéticas distintivas de las células leucémicas asociadas con el pronóstico en niños con leucemia linfoblástica aguda.*<br>***Definición operacional**: Se consignarán aquellas alteraciones genéticas reportadas positivas en estudios de citogenética por PCR en el expediente tras el diagnóstico de leucemia, dividiéndolas en los grupos siguientes:*<br>– *t(9;22)*<br>– *t(4;11)*<br>– *t(1:19)*<br>– *t(8;21) AML1-ETO*<br>– *t(15;17) PLM-RAR*<br>***Tipo de variable**: Cualitativa*<br>***Escala de medición**: Nominal politómica.*
Estatus de líquido cefalorraquídeo	***Definición conceptual**: La presencia de células leucémicas en líquido cefalorraquídeo.*<br>***Definición operacional**: Se recogerá el resultado obtenido del análisis de las muestras de punción lumbar al momento del diagnóstico y al finalizar la inducción a la remisión. Se clasificará de la siguiente manera el resultado:*<br>– *Positivo: presencia de células leucémicas en una punción lumbar no traumática.*<br>– *Hemorrágico: cuando se documenta una hemorragia interna por la evolución de la enfermedad.*<br>– *Traumático: cuando como resultado del procedimiento de punción lumbar se obtiene LCR sanguinolento.*<br>– *Negativo: ausencia de blastos.*<br>***Tipo de variable**: Cualitativa*<br>***Escala de medición**: Nominal politómica.*
Remisión	***Definición conceptual**: La reducción de la carga de células leucémicas en médula ósea por debajo de niveles morfológicamente detectables con recuperación de la hematopoyesis normal.*<br>***Definición operacional**: Se considerará remisión completa cuando los*

	registros del paciente cumpla las siguientes condiciones:<br>– *Aspirado de médula ósea < 5% de blastos*<br>– *Ausencia de blastos en líquido cefalorraquídeo*<br>– *Neutrófilos ≥1.5 x10^9/L*<br>– *Plaquetas >100 x10^9/L*<br>*Reportados el día 14, 21, 28 o 35 de la inducción.*<br>***Tipo de variable**: Cualitativa*<br>***Escala de medición**: Nominal dicotómica (en remisión/ sin remisión)*
Comorbilidades	**Definición conceptual:** Padecimientos concomitantes en el paciente al momento del diagnóstico de leucemia que no son complicaciones de la misma.<br>**Definición operacional:** Se contabilizará el número de afecciones antecedentes al diagnóstico de LLA, que fueron registradas en la base de datos de la cohorte, y que provienen de la historia clínica elaborada durante la primera hospitalización para diagnóstico. El número de comorbilidades será agrupado de la siguiente manera:<br>– Sin comorbilidades (0 antecedentes)<br>– Con comorbilidades (uno o más antecedentes independientes)<br>**Escala de medición:** Cualitativa.<br>**Escala de medición:** Nominal dicotómica.
Puntaje Z del índice de masa corporal	**Definición conceptual**: Razón entre el peso y la talla que se transforma en desviaciones estándar (DE) de la distribución Z por edad.<br>**Definición operacional:** se calculará el índice de masa corporal (IMC) transformado en desviaciones estándar del puntaje Z de cada paciente utilizando el software WHO AnthroPlus. Para su análisis se utilizarán las variables: edad (meses), sexo, peso (kg), talla (cm), fecha de diagnóstico y fecha de nacimiento. Los valores en escala continua calculados por AntroPlus serán agrupados de la siguiente manera:<br>– Desnutrición: < -1 DE<br>– Normopeso: -1 ≥ z ≤ +1 DE<br>– Sobrepeso: +2 ≥ z > +1 DE<br>– Obesidad: > +2 DE<br>**Tipo de variable:** Cualitativa.<br>**Escala de medición:** Ordinal.
Respuesta al corticosteroide	**Definición conceptual**: Reducción de la carga de blastos en sangre periférica.<br>**Definición operativa**: Se tomará el registro tomado de los expedientes clínicos en el día 8 de recibir la ventana de esteroides (dexametasona o prednisona). En base a la biometría hemática realizada ese día, el resultado podrá ser:<br>– Buena respuesta: < 1000 blastos en sangre periférica<br>– Mala respuesta: ≥ 1000 blastos en sangre periférica<br>– Incierto: en caso de no haber biometría hemática disponible.<br>**Tipo de variable**: Cualitativa.<br>**Escala**: Nominal.
Número de	**Definición conceptual**: Número de ingresos hospitalarios por EA durante la

hospitalizaciones	terapia de inducción a la remisión.<br>**Definición operativa**: Se contabilizará cada reingreso hospitalario por EA con una duración mayor a 24 horas durante la inducción a la remisión. Los reingresos por complicaciones originadas en hospitalizaciones previas serán contabilizados de manera independiente.<br>**Tipo de variable**: Cuantitativa de razón.<br>**Escala**: Discreta (ingresos hospitalarios).
Duración de la estancia hospitalaria	**Definición conceptual**: Tiempo transcurrido entre el ingreso y egreso de hospitalización.<br>**Definición operativa**: Se contabilizará el número de días que transcurren entre los ingresos y egresos de hospitalización mientras el paciente se encuentre bajo manejo médico en la fase de inducción a la remisión. La suma de los días de todos los ingresos hospitalarios dará la duración total de la estancia hospitalaria en esta fase de tratamiento.<br>**Tipo de variable**: Cuantitativa de razón.<br>**Escala**: Discreta (días).
Duración de la inducción a la remisión.	**Definición conceptual**: Tiempo que transcurre entre el inicio de la venta de esteroides y el final de la inducción a la remisión.<br>**Definición operativa**: Se contabilizará el número de días que transcurren desde el día en que el paciente recibe la primera dosis de esteroide (dexametasona o prednisona) hasta la fecha en que recibe la última dosis de vincristina y L-asparaginasa ordenada por el médico tratante.<br>**Tipo de variable**: Cuantitativa de razón.<br>**Escala**: Discreta (días).

Procedimiento.

Base de datos de la cohorte original.

La revisión de los expedientes clínicos para la construcción de esta cohorte se realizó entre 2018 y 2019 como parte del estudio HIM-2018-018[70]. Se obtuvieron los listados de pacientes diagnosticados con LLA entre enero de 2015 y diciembre de 2017 en el Departamento de Bioestadística del HIMFG.

La base de datos contiene variables demográficas de los pacientes, historia clínica previa, resultados del proceso diagnóstico de LLA y de evolución clínica con resultados de pruebas clínicas y paraclínicas realizadas durante la inducción a la remisión, además contiene variables que describen del manejo farmacológico quimioterapéutico y no quimioterapéutico: nombre del medicamento, posología, dosis, vías de administración, periodicidad, fechas de prescripción y

administración y la duración *del protocolo de inducción a la remisión del HIMFG, el cual está basado en el Protocolo XIII del San Jude Hospital*[83], así como el análisis de errores de medicación en las etapas de prescripción, transcripción y administración de los medicamentos. También incluye fechas de ingreso y egreso de cada hospitalización, junto con las causas de los reingresos hospitalarios (esto es, medicamentos, procedimientos médicos, cuidados en hospitalizaciones previas) se documentaron tan exhaustivamente cómo fue posible dada la naturaleza retrospectiva de la cohorte original, de tal manera que se cuenta con resultados de pruebas de laboratorio, observaciones en notas de interconsulta de especialidades, notas de enfermería, notas de evaluación médica, y medicación potencialmente asociada con EA, desde el primer ingreso al hospital para diagnóstico, hasta el final de la terapia de inducción a la remisión o fallecimiento del paciente. En caso de fallecimiento se cuenta con datos del certificado o acta de defunción anexo al expediente donde se especifican causas primarias y secundarias de fallecimiento, así como la fecha de muerte.

Identificación de los eventos adversos.

Los investigadores responsables del estudio invitarán de manera formal e independiente a dos oncólogos pediatras expertos en la atención de pacientes dentro del HIMFG, para participar como revisores expertos en la evaluación de EA, explicando los objetivos y alcances del estudio. Al aceptar participar en la revisión de los casos, se organizará una sesión de capacitación e información con cada oncólogo pediatra para ser capacitados en la aplicación de las escalas mencionadas para este estudio, es decir: la taxonomía CTCAE versión cinco para determinar el grado de severidad de los EA, así como los criterios de Gandhi y sus categorías de prevenibilidad. En esta sesión se resolverán dudas sobre el protocolo de estudio y se establecerán las vías de intercambio de información y comunicación con los investigadores responsables.

Para la identificación de los EA contenidos en la base de datos, se elaborarán resúmenes de cada paciente incluido en la cohorte describiendo el manejo de eventos médicos documentados, así como información sobre el tratamiento farmacológico administrado, evaluación de errores de medicación, diagnósticos de ingreso hospitalario y la duración de cada hospitalización, procedimientos quirúrgicos realizados, así como acciones médicas registradas en la base de datos, desde el ingreso hasta el final del seguimiento en la cohorte. Tales resúmenes se harán llegar vía electrónica a los revisores expertos, quienes evaluarán de manera independientemente los casos enviados.

Evaluación independiente por expertos.

Los revisores expertos emitirán un juicio sobre la presencia o ausencia de EA asociados al cuidado de la salud durante el manejo de inducción a la remisión. En todos los casos donde emitan un juicio afirmativo a la presencia de uno o más EA, se indicará la causa más probable. Para esto, los revisores expertos podrán pedir mayor información sobre periodos o eventos específicos de la evolución clínica de cualquier caso enviado. Los investigadores serán los responsables de enviar la información requerida, en caso de no existir más información que proporcionar se informará a los revisores para que emitan su juicio.

Una vez que los revisores clasifiquen los EA en base a la terminología CTCAE deberán elegir la severidad del evento en base a los criterios CTCAE y su prevenibilidad con los criterios de Gandhi. Una vez evaluados los casos, los revisores expertos enviarán de vuelta vía correo electrónico sus resultados. Los investigadores responsables descargarán las variables de presencia de EA, tipo de EA, severidad y prevenibilidad con el grado asignado por los revisores en una sola base de datos en una hoja de cálculo de Microsoft Excel que identifique con una clave al paciente que pertenecen. Los datos serán ordenados y se medirá el grado de concordancia entre evaluadores. Aquellos EA donde los revisores no coincidieron en la evaluación de la primera

etapa serán discutidos hasta alcanzar un consenso, con la mediación del investigador responsable por video llamada. En caso de no alcanzar un consenso, uno de los investigadores tomará la decisión actuando como tercer revisor.

Diseño estadístico.

Verificación de registros.

Análisis descriptivo. Las variables cualitativas se expresarán como frecuencias relativas y porcentajes. Por otro lado, se estudiarán las distribuciones de las variables cuantitativas a través de la asimetría (-1 a +1) y la curtosis (-1 a +1), obteniendo así estimaciones de tendencia central y dispersión: media ($\bar{x}$) y desviación estándar para variables continuas con distribución normal, y mediana (me) y rango intercuartílico (RI) para variables con distribución libre. No se imputarán datos faltantes.

Confiabilidad entre revisores. Se utilizará la prueba de kappa de Cohen (k) para evaluar el acuerdo entre los revisores expertos en la determinación de EA. Además, se utilizará la prueba de kappa ponderada (k_w) para medir el grado de acuerdo al asignar el grado de severidad, y prevenibilidad de los EA identificados. Los tres coeficientes resultantes serán expresados como porcentaje.

Estimación de tasas de incidencia. Se estimarán las tasas de incidencia de los EA por tipo, severidad, y prevenibilidad cada 1000 días-paciente y cada 1000 días-paciente en hospitalización durante la inducción remisión. Para esto, se contará el número total de EA, así como el número de EA originados dentro del hospital. Así también, se contarán los días-paciente acumulados desde el inicio del abordaje diagnóstico hasta el final de la inducción o el fallecimiento del paciente, así como los días-paciente transcurridos en hospitalización. Adicionalmente, se estimarán las proporciones de EA por tipo, severidad y prevenibilidad cada 100 pacientes en

inducción a la remisión y por cada 100 hospitalizaciones en el mismo periodo. Para todos los estimadores de incidencia se calcularán intervalos de confianza al 95%.

Todos los análisis estadísticos se realizarán con el software IBM SPSS Statistics versión 25.

Consideraciones éticas y de bioseguridad

Ética. Este estudio comprende el análisis y la evaluación secundaria de una cohorte de pacientes pediátricos con LLA del HIMFG, construida a partir de fuentes secundarias de documentación (expediente clínico), de manera observacional como parte del protocolo HIM-2018-018, "Evaluación de Errores de Medicación durante el Tratamiento de Inducción a la Remisión en Pacientes Pediátricos con Leucemia Linfoblástica Aguda", el cual contó con la aprobación de los Comités de Investigación, Ética y Bioseguridad del HIMFG como un estudio "Sin Riesgo" para los pacientes. Por lo anterior, se pone a consideración del Comité de Ética en Investigación la dispensación del Consentimiento Informado de los pacientes cuya información será analizada en este proyecto de investigación. Es importante mencionar que los investigadores no difundirán información que identifique a los pacientes como: nombres, iniciales o el número de registro institucional. Adicionalmente, las RAMs identificadas en este estudio como EAM se reportarán al Centro Nacional de Farmacovigilancia a través de la plataforma Vigiflow, de acuerdo con la Norma Oficial Mexicana NOM-2020-SSA1-2016, instalación y operación de la farmacovigilancia.

Bioseguridad. De acuerdo a las instrucciones de llenado del Formato de Bioseguridad consideramos que este protocolo "No tiene implicaciones de Bioseguridad".

REFERENCIAS

1. Quality of care. World Health Organization. [Citado abril 21, 2021]. Disponible en: https://www.who.int/health-topics/quality-of-care#tab=tab_1
2. Campbell SM, Roland MO, Buetow SA. Defining quality of care. Soc Sci Med. 2000;51(11):1611-25.
3. Draft Global Patient Safety Action Plan 2021–2030. Towards eliminating avoidable harm in health care. World Health Organization-Patienti Safety. [Citado abril 21, 2021]. Disponible en: https://cdn.who.int/media/docs/default-source/patient-safety/gpsap/global-patient-safety-action-plan_12-apr-2021.pdf?sfvrsn=58ad8e9e_10
4. Patient safety. World Health Organization. [Citado abril 21, 2021]. Disponible en: https://www.who.int/teams/integrated-health-services/patient-safety
5. National Academies of Sciences, Engineering, and Medicine; Health and Medicine Division; Board on Health Care Services; Board on Global Health; Committee on Improving the Quality of Health Care Globally. Crossing the global quality chasm: improving health care worldwide. Washington (DC): National Academies Press; 2018.
6. NHS Litigation Authority. Annual report and accounts 2017/18. London: Her Majesty's Stationery Office; 2018. [Citado abril 21, 2021]. Disponible en: https://resolution.nhs.uk/wp-content/uploads/2018/08/NHSResolution-Annual-Report-2017-2018.pdf
7. Hiatt HH, Barnes BA, Brennan TA, Laird NM, Lawthers AG, Leape LL, et al. A study of medical injury and medical malpractice. N Engl J Med. 1989;321(7):480-484.
8. Brennan TA, Leape LL, Laird NM, Hebert L, Localio AR, Lawthers AG, et al. Incidence of adverse events and negligence in hospitalized patients. Results of the Harvard Medical Practice Study I. N Engl J Med. 1991;324(6):370-376.
9. World alliance for patient safety: WHO draft guidelines for adverse event reporting and learning systems: from information to action. World Health Organization; 2005. [Citado junio 14, 2021]. Disponible en: https://apps.who.int/iris/handle/10665/69797

10. McFatrich M, Brondon J, Lucas NR, Hinds PS, Maurer SH, Mack JW, et al. Mapping child and adolescent self-reported symptom data to clinician-reported adverse event grading to improve pediatric oncology care and research. Cancer. 2020;126(1):140-147.

11. Nebeker JR, Barach P, Samore MH. Clarifying adverse drug events: a clinician's guide to terminology, documentation, and reporting. Ann Intern Med. 2004;140(10):795-801.

12. Lipitz-Snyderman A, Pfister D, Classen D, Atoria CL, Killen A, Epstein AS, et al. Preventable and mitigable adverse events in cancer care: Measuring risk and harm across the continuum. Cancer. 2017;123(23):4728-36.

13. Rodziewicz TL, Houseman B, Hipskind JE. Medical Error Reduction and Prevention. [Actualizado enero 4, 2021]. En: StatPearls [Internet]. Treasure Island (FL): StatPearls Publishing; 2021. Disponible en: https://www.ncbi.nlm.nih.gov/books/NBK499956/

14. Weingart SN, Nelson J, Koethe B, Yaghi O, Dunning S, Feldman A, et al. Developing a cancer-specific trigger tool to identify treatment-related adverse events using administrative data. Cancer Med. 2020;9(4):1462-72.

15. de Vries EN, Ramrattan MA, Smorenburg SM, Gouma DJ, Boermeester MA. The incidence and nature of in-hospital adverse events: a systematic review. Qual Saf Health Care. 2008;17(3):216-23.

16. Williams DJ, Olsen S, Crichton W, Witte K, Flin R, Ingram J, et al. Detection of adverse events in a Scottish hospital using a consensus-based methodology. Scott Med J. 2008;53(4):26-30.

17. Naessens JM, O'Byrne TJ, Johnson MG, Vansuch MB, McGlone CM, Huddleston JM. Measuring hospital adverse events: assessing inter-rater reliability and trigger performance of the Global Trigger Tool. Int J Qual Health Care. 2010;22(4):266-74.

18. Matlow AG, Cronin CM, Flintoft V, Nijssen-Jordan C, Fleming M, Brady-Fryer B, et al. Description of the development and validation of the Canadian Paediatric Trigger Tool. BMJ Qual Saf. 2011;20(5):416-23.

19. Alghamdi AA, Keers RN, Sutherland A, Ashcroft DM. Prevalence and Nature of Medication Errors and Preventable Adverse Drug Events in Paediatric and Neonatal Intensive Care Settings: A Systematic Review. Drug Saf. 2019;42(12):1423-36.

20. Woo SA, Cragg A, Wickham ME, Peddie D, Balka E, Scheuermeyer F, et al. Methods for evaluating adverse drug event preventability in emergency department patients. BMC Med Res Methodol. 2018;18(1):160. doi:10.1186/s12874-018-0617-4

21. Salaets T, Turner MA, Short M, Ward RM, Hokuto I, Ariagno RL, et al. Development of a neonatal adverse event severity scale through a Delphi consensus approach. Arch Dis Child. 2019;104(12):1167-73.

22. Schwendimann R, Blatter C, Dhaini S, Simon M, Ausserhofer D. The occurrence, types, consequences and preventability of in-hospital adverse events - a scoping review. BMC Health Serv Res. 2018;18(1):521. doi: 10.1186/s12913-018-3335-z

23. Organisation for Economic Co-operation and Development. Measuring Patient Safety: Opening the Black Box. Paris, France: OECD; 2018. [Citado 14 junio, 2021]. Disponible en: https://www.oecd.org/health/health-systems/Measuring-Patient-Safety-April-2018.pdf

24. WHO guidelines for safe surgery 2009: safe surgery saves lives. Ginebra: Organización Mundial de la Salud; 2009. [Citado abril 22, 2021]. Disponible en: http://apps.who.int/iris/bitstream/handle/10665/44185/9789241598552_eng.pdf?sequence=1

25. Echevarría ZS, Sandoval CF, Gutiérrez DS, Alcantar BA, Cote EL. Eventos adversos en cirugía. Cir. gen [revista en la Internet]. 2011 ;33(3):163-169. [citado junio 14, 2021]. Disponible en: http://www.scielo.org.mx/scielo.php?script=sci_arttext&pid=S1405-00992011000300005&lng=es.

26. Gawande AA, Thomas EJ, Zinner MJ, Brennan TA. The incidence and nature of surgical adverse events in Colorado and Utah in 1992. Surgery. 1999;126(1):66-75.

27. Anderson O, Davis R, Hanna GB, Vincent CA. Surgical adverse events: a systematic review. Am J Surg. 2013;206(2):253-62.

28. Boeker EB, de Boer M, Kiewiet JJ, Lie-A-Huen L, Dijkgraaf MG, Boermeester MA. Occurrence and preventability of adverse drug events in surgical patients: a systematic review of literature. BMC Health Serv Res. 2013;13:364. doi: 10.1186/1472-6963-13-364.

29. Bates DW, Cullen DJ, Laird N, Petersen LA, Small SD, Servi D, et al. Incidence of adverse drug events and potential adverse drug events. Implications for prevention. ADE Prevention Study Group. JAMA. 1995;274(1):29-34.

30. Woo SA, Cragg A, Wickham ME, Villanyi D, Scheuermeyer F, Hau JP, et al. Preventable adverse drug events: Descriptive epidemiology. Br J Clin Pharmacol. 2020;86(2):291-302.

31. Una atención limpia es una atención más segura. Carga mundial de infecciones asociadas a la atención sanitaria. [Internet]. Organización Mundial de la Salud. [Citado mayo 21, 2021]. Disponible en: https://www.who.int/gpsc/country_work/burden_hcai/es/#:~:text=Las%20IAAS%2C%20tambi%C3%A9n%20denominadas%20infecciones,el%20momento%20de%20su%20ingreso.

32. Horan TC, Andrus M, Dudeck MA. CDC/NHSN surveillance definition of health care-associated infection and criteria for specific types of infections in the acute care setting. Am J Infect Control. 2008;36(5):309-32.

33. Report on the burden of endemic health care-associated infection worldwide. Ginebra: Organización Mundial de la Salud; 2011. [Citado abril 22, 2021]. Disponible en: http://apps.who.int/iris/bitstream/handle/10665/80135/9789241501507_eng.pdf?sequence=1

34. Khan LM. Comparative epidemiology of hospital-acquired adverse drug reactions in adults and children and their impact on cost and hospital stay--a systematic review. Eur J Clin Pharmacol. 2013;69(12):1985-96.

35. NCC MERP Index for Categorizing Medication Errors. National Coordinating Council for Medication Error Reporting and Prevention. [Citado mayo 22, 2021]. Disponible en: https://www.nccmerp.org/types-medication-errors.

36. Common Terminology Criteria for Adverse Events (CTCAE) v5.0. [Actualizado mayo 27, 2020]. In: Cancer Therapy Evaluation Program (CTEP) [Internet]. National Institutes of Health: U.S. Department of Health and Human Services; 2017. [Citado junio 1,

2021]. Disponible en: https://ctep.cancer.gov/protocolDevelopment/electronic_applications/docs/CTCAE_v5_Quick_Reference_5x7.pdf

37. Call RJ, Burlison JD, Robertson JJ, Scott JR, Baker DK, Rossi MG, et al. Adverse drug event detection in pediatric oncology and hematology patients: using medication triggers to identify patient harm in a specialized pediatric patient population. J Pediatr. 2014;165(3):447-52.e4. Doi: 10.1016/j.jpeds.2014.03.033.

38. Hébert G, Netzer F, Kouakou SL, Lemare F, Minvielle E; IATRIGGER Working Group. Development of a 'ready-to-use' tool that includes preventability, for the assessment of adverse drug events in oncology. Int J Clin Pharm. 2018;40(2):376-85.

39. West Z, Allen K, Monroe C, Castellino SM, Miller TP. Adverse events during induction therapy in pediatric acute lymphoblastic lymphoma (ALL). Blood. 2019;134(Supplement_1):5809. doi: 10.1182/blood-2019-131873

40. Kirkendall ES, Kloppenborg E, Papp J, White D, Frese C, Hacker D, et al. Measuring adverse events and levels of harm in pediatric inpatients with the Global Trigger Tool. Pediatrics. 2012;130(5):e1206-14. doi: 10.1542/peds.2012-0179.

41. Hakkarainen KM, Andersson Sundell K, Petzold M, Hägg S. Methods for assessing the preventability of adverse drug events: a systematic review. Drug Saf. 2012;35(2):105-126.

42. Leape LL, Lawthers AG, Brennan TA, Johnson WG. Preventing medical injury. QRB Qual Rev Bull. 1993;19(5):144-9.

43. Gandhi TK, Weingart SN, Borus J, Seger AC, Peterson J, Burdick E, et al. Adverse drug events in ambulatory care. N Engl J Med. 2003;348(16):1556-64.

44. Pronovost PJ, Colantuoni E. Measuring preventable harm: helping science keep pace with policy. JAMA. 2009;301(12):1273-5.

45. Pérez FGM. Responsabilidad civil médica y la aplicación de los daños punitivos en México. Rev. IUS [Revista en internet]. 2020;14(46):143-62. [citado mayo 24, 2021]. Disponible en: <http://www.scielo.org.mx/scielo.php?script=sci_arttext&pid=S1870-21472020000200143&lng=es&nrm=iso>. Epub 02-Dic-2020. ISSN 1870-2147.

46. Ferner RE, Aronson JK. Clarification of terminology in medication errors: definitions and classification. Drug Saf. 2006;29(11):1011-22.

47. Dubois RW, Brook RH. Preventable deaths: who, how often, and why? Ann Intern Med. 1988;109(7):582-9.

48. Hallas J, Harvald B, Gram LF, Grodum E, Brøsen K, Haghfelt T, et al. Drug related hospital admissions: the role of definitions and intensity of data collection, and the possibility of prevention. J Intern Med. 1990;228(2):83-90.

49. Schumock GT, Thornton JP. Focusing on the preventability of adverse drug reactions. Hosp Pharm. 1992;27(6):538.

50. Rivera-Luna R, Shalkow-Klincovstein J, Velasco-Hidalgo L, Cárdenas-Cardós R, Zapata-Tarrés M, Olaya-Vargas A, et al. Descriptive epidemiology in Mexican children with cancer under an open national public health insurance program. BMC Cancer. 2014;14:790. doi: 10.1186/1471-2407-14-790.

51. Stewart BW, Wild CP, eds. World Cancer Report 2014. Lyon, France: World Health Organization Press. 2014.

52. Cooper SL, Brown PA. Treatment of pediatric acute lymphoblastic leukemia. Pediatr Clin North Am. 2015;62(1):61–73.

53. Magrath I, Steliarova-Foucher E, Epelman S, Ribeiro RC, Harif M, Li CK, et al. Paediatric cancer in low-income and middle-income countries. Lancet Oncol. 2013;14(3):e104-16. doi: 10.1016/S1470-2045(13)70008-1.

54. Rendón-Macías ME, Reyes-Zepeda NC, Villasís-Keever MA, Serrano JM, Escamilla AN. Tendencia mundial de la supervivencia en pacientes pediátricos con leucemia linfoblástica aguda. Revisión de las últimas cuatro décadas. Bol Med Hosp Infant Mex. 2012;69(3):153-63.

55. Aguilar-Hernández M, Fernández-Castillo G, Nuñez-Villegas NN, Pérez-Casillas RX, Núñez-Enríquez JC. Principales causas de mortalidad durante la fase de inducción en los pacientes pediátricos con leucemia linfoblástica aguda. Rev Med Inst Mex Seguro Soc. 2017;55(3):286-91.

56. Rivera-Luna R, Olaya-Vargas A, Velásquez-Aviña M, Frenk S, Cárdenas-Cardós R, Leal-Leal C, et al. Early death in children with acute lymphoblastic leukemia: does malnutrition play a role? Pediatr Hematol Oncol. 2008;25(1):17-26. doi: 10.1080/08880010701774132.

57. Hunger SP, Mullighan CG. Acute Lymphoblastic Leukemia in Children. N Engl J Med. 2015;373(16):1541-52.

58. Pui CH, Evans WE. Treatment of Acute Lymphoblastic Leukemia. N Engl J Med. 2006; 354: 166-78.

59. Baytan B, Evim MS, Güler S, Güneş AM, Okan M. Acute Central Nervous System Complications in Pediatric Acute Lymphoblastic Leukemia. Pediatr Neurol. 2015;53(4):312-8.

60. Li MJ, Chang HH, Yang YL, Lu MY, Shao PL, Fu CM, et al. Infectious complications in children with acute lymphoblastic leukemia treated with the Taiwan Pediatric Oncology Group protocol: A 16-year tertiary single-institution experience. Pediatr Blood Cancer. 2017;64(10). doi: 10.1002/pbc.26535.

61. Barajas-Nava LA, Garduño-Espinosa J. Medical interventions for cancer treatment-induced symptoms in children: an overview. Bol Med Hosp Infant Mex. 2016;73(6):467-83.

62. Weaver MS, Reeve BB, Baker JN, Martens CE, McFatrich M, Mowbray C, et al. Concept-elicitation phase for the development of the pediatric patient-reported outcome version of the Common Terminology Criteria for Adverse Events. Cancer. 2016;122(1):141-8.

63. Howard SC, Davidson A, Luna-Fineman S, Israels T, Chantada G, Lam CG, et al. A framework to develop adapted treatment regimens to manage pediatric cancer in low- and middle-income countries: The Pediatric Oncology in Developing Countries (PODC) Committee of the International Pediatric Oncology Society (SIOP). Pediatr Blood Cancer. 2017;64(Suppl 5). doi: 10.1002/pbc.26879.

64. Houweling TA, Kunst AE. Socio-economic inequalities in childhood mortality in low- and middle-income countries: a review of the international evidence. Br Med Bull. 2010;93:7-26.

65. Pui CH. Reducing delayed intensification therapy in childhood ALL. Lancet Oncol. 2013;14(3):178-9.

66. Higashiyama Y, Kojima C, Kubota M, et al. Longitudinal nutritional assessment in acute lymphoblastic leukemia during treatment. Pediatr Int. 2014;56(4):541-46.

67. Morales-Ríos O, Cicero-Oneto C, García-Ruiz C, Villanueva-García D, Hernández-Hernández M, Olivar-López V, et al. Descriptive study of adverse drug reactions in a tertiary care pediatric hospital in México from 2014 to 2017. PLoS One. 2020;15(3):e0230576. doi: 10.1371/journal.pone.0230576.

68. Gerber A, Da Silva Lopes A, Szüts N, Simon M, Ribordy-Baudat V, Ebneter A, et al. Describing adverse events in Swiss hospitalized oncology patients using the Global Trigger Tool. Health Sci Rep. 2020;3(2):e160. doi: 10.1002/hsr2.160.

69. Edwards IR, Aronson JK. Adverse drug reactions: definitions, diagnosis, and management. Lancet. 2000;356(9237):1255-59.

70. Vázquez-Cornejo E, Morales-Ríos O, Juárez-Villegas LE, Islas Ortega EJ, Vázquez-Estupiñán F, Garduño-Espinosa J. Medication errors in a cohort of pediatric patients with acute lymphoblastic leukemia on remission induction therapy in a tertiary care hospital in Mexico. Cancer Med. 2019;8(13):5979-87.

71. Nazer LH, Hawari F, Al-Najjar T. Adverse drug events in critically ill patients with cancer: incidence, characteristics, and outcomes. J Pharm Pract. 2014;27(2):208-13.

72. Nazer LH, Eljaber R, Rimawi D, Hawari FI. Adverse drug events resulting in admission to the intensive care unit in oncology patients: Incidence, characteristics and associated cost. J Oncol Pharm Pract. 2013;19(4):298-304.

73. Chopra D, Rehan HS, Sharma V, Mishra R. Chemotherapy-induced adverse drug reactions in oncology patients: A prospective observational survey. Indian J Med Paediatr Oncol. 2016;37(1):42-46.

74. Lau PM, Stewart K, Dooley M. The ten most common adverse drug reactions (ADRs) in oncology patients: do they matter to you?. Support Care Cancer. 2004;12(9):626-33.

75. Shrestha S, Shakya R, Shrestha S, Shakya S. Adverse Drug Reaction due to Cancer Chemotherapy and its Financial Burden in Different Hospitals of Nepal. Int J Pharmacovigil. 2017;2(1):1-7.

76. Wahlang JB, Laishram PD, Brahma DK, Sarkar C, Lahon J, Nongkynrih BS. Adverse drug reactions due to cancer chemotherapy in a tertiary care teaching hospital. Ther Adv Drug Saf. 2017;8(2):61-6.

77. Ramasubbu SK, Pasricha RK, Nath UK, Das B. Frequency, nature, severity and preventability of adverse drug reactions arising from cancer chemotherapy in a teaching hospital. J Family Med Prim Care. 2020;9(7):3349-55.

78. Sharma A, Kumari KM, Manohar HD, Bairy KL, Thomas J. Pattern of adverse drug reactions due to cancer chemotherapy in a tertiary care hospital in South India. Perspect Clin Res. 2015;6(2):109-15.

79. Berchialla P, Scaioli G, Passi S, Gianino MM. Adverse events in hospitalized paediatric patients: a systematic review and a meta-regression analysis. J Eval Clin Pract. 2014;20(5):551-8.

80. Stockwell DC, Landrigan CP, Toomey SL, Loren SS, Jang J, Quinn JA, Ashrafzadeh S, Wang MJ, Wu M, Sharek PJ, Classen DC, Srivastava R, Parry G, Schuster MA; GAPPS Study Group. Adverse Events in Hospitalized Pediatric Patients. Pediatrics. 2018;142(2):e20173360. doi: 10.1542/peds.2017-3360.

81. Leape LL, Brennan TA, Laird N, Lawthers AG, Localio AR, Barnes BA, et al. The nature of adverse events in hospitalized patients. Results of the Harvard Medical Practice Study II. N Engl J Med. 1991;324(6):377-84.

82. Soop M, Fryksmark U, Köster M, Haglund B. The incidence of adverse events in Swedish hospitals: a retrospective medical record review study. Int J Qual Health Care. 2009;21(4):285-91

83. Protocolo de manejo para pacientes pediátricos con leucemia linfoblástica no tratados previamente. En Guías [Internet]. Hospital Infantil de México Federico Gómez. [Citado agosto 25, 2021]. Disponible en: http://www.himfg.edu.mx/descargas/documentos/planeacion/guiasclinicasHIM/LeuAguLinfoblastica.pdf

Investigadores.

Investigador responsable.

Edmundo Vázquez Cornejo

Departamento de Evaluación de Medicamentos y Farmacovigilancia

55 5228 9917 ext. 2356.

edmundovaco21@gmail.com

Investigador suplente.

Olga Magdala Morales Ríos

Departamento de Evaluación de Medicamentos y Farmacovigilancia/Unidad Habilitada de Apoyo al Predictamen.

55 5228 9917 ext. 2356.

magdalaqfb@yahoo.com.mx

Investigadores asociados.

Carlo Cicero Oneto

Departamento de hemato-oncología.

Gabriela Hernández Pliego

Departamento de hemato-oncología.

Juan Garduño Espinosa

Dirección de Investigación.
