# Supplementary material for: Incidence, severity, and preventability of adverse events during the induction of patients with acute lymphoblastic leukemia in a tertiary care pediatric hospital in Mexico
Source: PLoS One. 2022 Mar 24;17(3):e0265450. doi: 10.1371/journal.pone.0265450 (PMC8947076; doi:10.1371/journal.pone.0265450)
Supplement: S2 File — (PDF) [file pone.0265450.s010.pdf]

Ciudad de México, 23 de septiembre del 2021

DG/1000/ 750 /2021

M en C. Edmundo Vázquez Cornejo
Unidad Habilitada de Apoyo al Predictamen
Presente

Informo a usted, que los Comités de Investigación, Ética en Investigación y Bioseguridad, después de haber revisado su protocolo **HIM-2021-065** titulado **"Incidencia, severidad y prevenibilidad de eventos adversos en el tratamiento de inducción a la remisión de pacientes con leucemia linfoblástica aguda en un hospital pediátrico de tercer nivel en México",** han emitido el dictamen de:

APROBADO

En los términos y condiciones señalados por dichos Comités. Por lo anterior, se autoriza su desarrollo.

Atentamente

Dr. Jaime Nieto Zermeño
Director General

Con copia:
Dr. Juan Garduño Espinosa. Director de Investigación.

JNZ/JGE/MSG/vzn
